# Overcoming the Druggability Hurdles of Celastrol: A Critical Review of Advanced Drug Delivery Strategies

**DOI:** 10.3390/biom16070932

**Published:** 2026-06-23

**Authors:** Keren Xu, Yue Wang, Hong Wang, Xuanrong Sun, Zhikun Yang

**Affiliations:** 1 College of Pharmaceutical Science & Jianxing Honors College, Zhejiang University of Technology, Hangzhou 310014, China; 2 Zhejiang-Egypt Joint Laboratory on Intelligent Discovery of Marine Drugs, Hangzhou 310014, China; 3 Zhejiang Key Laboratory of Green Manufacturing Technology for Chemical Drugs, Hangzhou 310014, China

**Keywords:** celastrol, delivery systems, nanoparticles, vesicles, emulsions

## Abstract

Celastrol, one of the top five traditional natural products with high potential for modern drug development, exerts potent broad-spectrum biological activities, yet its poor aqueous solubility, low bioavailability, potential toxicity, and limited selectivity severely compromise its drug-likeness. Advanced drug delivery strategies, mainly including multifunctional polymer/lipid/protein-based organic nanoparticles, metal/silica-based inorganic nanoparticles, vesicles represented by liposomes, and nanoemulsions, are expected to overcome these druggability hurdles of celastrol via oral, transdermal or intravenous administration. This review summarizes recent progress in a series of celastrol formulations, including novel dosage forms and delivery routes accompanied with consequential pharmacological effects and mechanisms of action, which have the potential to bring about better druggability conducive to future medical treatment.

## 1. Introduction

Celastrol, a friedelin-type pentacyclic triterpenoid bearing a highly conjugated quinone-methide moiety, is ubiquitously distributed in Celastraceae plant species. First isolated in 1936 from *Tripterygium wilfordii*, celastrol has garnered substantial research interest owing to its favorable low molecular weight paired with a structurally intricate polycyclic framework. Accumulating studies have demonstrated that celastrol possesses versatile biological activities, showing promising therapeutic potential against cancer, inflammation, metabolic disorders, neurodegenerative diseases, and cardiovascular and cerebrovascular diseases, as well as infectious diseases [1,2]. The unique structural features and multiple biological functions of celastrol, combined with its therapeutic potential, have driven global research interests in developing new drug leads based on celastrol. Despite this, the clinical translation of celastrol remains constrained by several physicochemical and pharmacokinetic challenges, including poor water solubility, low bioavailability, and inadequate target specificity. Meanwhile, its associated toxic risks and systemic adverse reactions are equally substantial [3]. Notable toxicities include cardiotoxicity [4,5,6,7], hepatotoxicity [7,8,9,10], gastrointestinal toxicity [10,11], nephrotoxicity [12], lipotoxicity [13], reproductive toxicity [14,15,16], hematopoietic toxicity [17], and other side effects [18], rendering celastrol a perilous compound with a narrow therapeutic window and significantly constraining its clinical application. Indubitably, further investigation into related problem-solving strategies is essential for improving its druggability.

**Figure 1:**
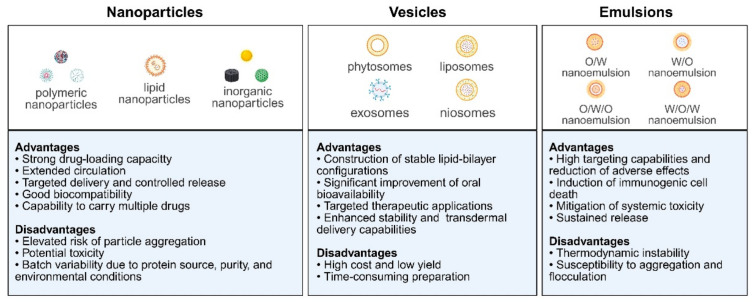
A short graphical overview comparing the major delivery platforms and their advantages/disadvantages.

Considering that celastrol has broad prospects for medicinal use, in-depth research on its targets and various methods to enhance efficacy and reduce toxicity are expected to promote its clinical application with a positive impact on disease treatment. To address the above issues, developing novel drug delivery systems through direct chemical modification or indirect physical encapsulation can enhance the selectivity of drug for target cells, thereby maintaining efficacy while minimizing toxicity. Researchers are continually developing novel drug formulations and technologies incorporating celastrol, leading to patentable medications with high loading capacity, controlled release, and improved stability and safety. These advancements are pivotal in expediting the modernization of monomer preparation processes (Figure 1). From different perspectives, several reviews have summarized key aspects of studies based on celastrol formulations in recent years, which are classified according to formulations, indications, and routes of administration, respectively [19,20,21,22]. This paper focuses on recent advances of celastrol delivery systems (2020–2024) and makes several corrections to previous reviews based on the classification of dosage forms in a more professional and authoritative reference [23]. Meanwhile, with the goal of informing and guiding future research efforts, comprehensive and detailed progresses in its physicochemical properties, pharmacological activities, and biological safety have also been further discussed, which are accompanied by a profound analysis of pros and cons, clinical translation obstacles, and future research directions.

## 2. Celastrol Delivery Systems

### 2.1. Nanoparticles

**Figure 2:**
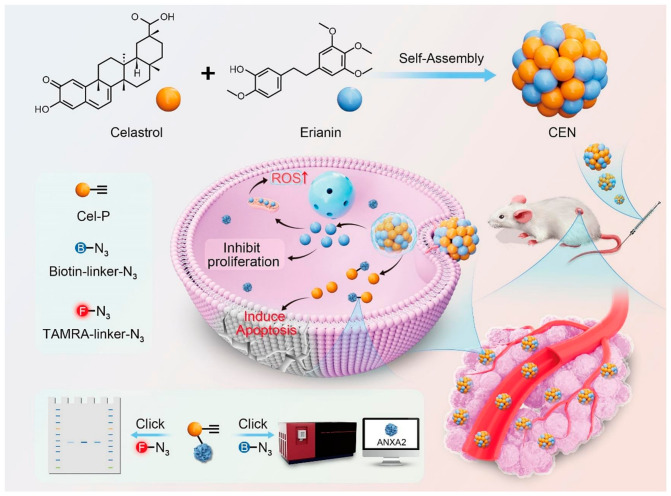
Schematic illustration of mechanism of self-assembled carrier-free celastrol (Cel)-erianin nanoparticles (CEN) for the treatment of breast cancer developed by Tian et al. CEN for breast cancer treatment were prepared by self-assembling natural compounds. Annexin A2 (ANXA2) was identified and validated as the direct target through which Cel in CEN induced apoptosis using the activity-based protein profiling (ABPP) method. CEN effectively inhibited the proliferation of breast cancer cells and induced apoptosis, providing a synergistic treatment for breast cancer. Reprinted with permission from reference [28]. Copyright 2024 Chemical Engineering Journal.

Nanoparticles, sized 1–100 nm, are notable for their strong drug-loading capacity, extended circulation, and targeted delivery capabilities, which can be achieved through size control, structural modification, and surface decoration [24]. They enhance drug solubility and can carry multiple drugs with controlled release, offering advantages over free drugs [25]. This is crucial for understanding drug–cell interactions and advancing clinical applications of nanocarriers. Recent developments in nano-drug delivery systems, integrating polymer materials and pharmacology, have revolutionized drug development and clinical use [26]. For instance, celastrol, when loaded onto nanocarriers, can effectively target tumors, improve solubility, and cross the blood–brain barrier, enhancing its therapeutic effects. The limited water solubility and lack of tumor specificity of celastrol present significant obstacles to its clinical application. To overcome these challenges, nano-formulation strategies based on self-assembly have been developed. These strategies enhance drug delivery and therapeutic efficacy while minimizing systemic toxicity. For example, celastrol can be formulated into carrier-free nanoparticles, which improve tumor accumulation and inhibit growth in breast cancer models [27]. Co-assembly with other agents, such as erianin, not only enhances solubility but also achieves synergistic anti-tumor effects by inducing cell cycle arrest and inhibiting metastasis (Figure 2) [28]. Additionally, to enhance active targeting, nanoparticles can be functionalized with specific ligands. For instance, a galactose-modified nanomedicine successfully targeted asialoglycoprotein receptors on hepatocellular carcinoma cells, inducing cell death through a reactive oxygen species (ROS)-mediated ferroptosis pathway [29]. Collectively, these self-assembled nanoarchitectures represent a potent approach to optimizing the therapeutic profile of celastrol for cancer treatment.

#### 2.1.1. Polymeric Nanoparticles

**Figure 3:**
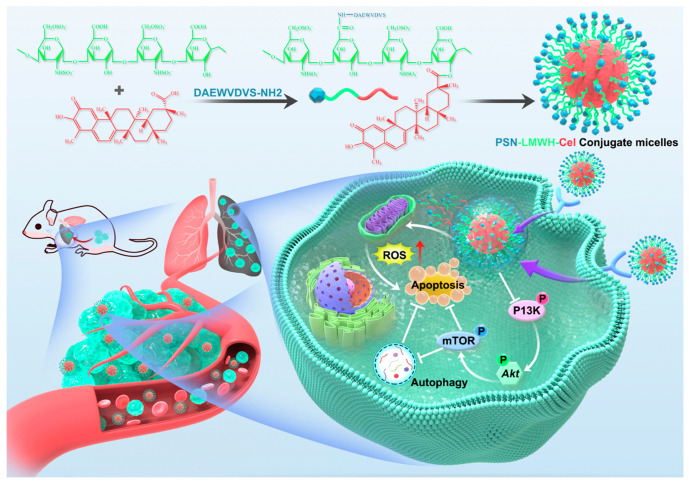
Schematic illustration of preparation and in vivo anticancer mechanism of conjugate nanodrugs (PLC-NP) using celastrol (Cel) developed by Zhou et al. A self-assembled nanodrug was designed by conjugating the antitumor drug Cel with the bioactive material low molecular weight heparin (LMWH), which served as hydrophilic material providing water solubility for polymers and affording colloidal stability for assembled nanoscale particles. In addition, LMWH synergized with Cel by targeting tumor vessels and inhibiting tumor angiogenesis. Since P-selectin targeting peptide (PSN) specifically bound to P-selectin which is highly expressed on most cancer cells and activated platelets at the tumor site, therefore, the optimized nano-drug was further decorated with PSN to enhance its cancer-targeting capability and efficacy. In vitro and in vivo experiments confirmed that PLC-NP efficiently decreased the adverse effects of severe toxicities of Cel on organs and blood, and exhibited excellent anti-tumor efficacy in different tumor mouse models. Reprinted with permission from reference [50]. Copyright 2023 eBioMedicine.

Polymeric nanoparticles, made from natural or synthetic materials, can be crafted using techniques like emulsification, nanoprecipitation, ionic gelation, and microfluidics, resulting in diverse structures and properties. They offer versatile drug delivery options, allowing encapsulation, entrapment or surface binding of therapeutics, including hydrophobic/hydrophilic compounds and various molecular weights like small molecules and vaccines. These nanoparticles are ideal for co-delivery, with modifiable properties such as composition and surface charge to control loading and release kinetics [30,31,32]. Polymer-based nanocarriers for celastrol are predominantly designed through the strategic selection of material origin, whether natural or synthetic, and the drug incorporation method, either encapsulation or conjugation. These choices collectively influence targeting specificity, stimuli-responsiveness, and therapeutic efficacy [33,34]. Natural polymers such as chitosan, zein, and *β*-cyclodextrin are frequently preferred due to their inherent biocompatibility, bioactivity, and ease of functionalization, which facilitate oral delivery and targeted specificity [35,36,37,38,39]. In contrast, synthetic polymers like poly(lactic-*co*-glycolic acid) (PLGA) and poly(ethylene glycol) (PEG) provide enhanced tunability of physicochemical properties, degradation kinetics, and the precise integration of stimuli-responsive elements. This enables the development of “smart” systems that can respond to pathological cues such as pH and ROS [40,41,42,43,44,45,46,47,48,49]. The strategy for drug loading further distinguishes the performance of carriers. While physical encapsulation is simpler, it may result in variable loading and release profiles due to its dependence on drug-polymer compatibility. In contrast, chemical conjugation using cleavable linkers, such as ester (Figure 3) and imine bonds, produces prodrug-based nanoparticles that offer enhanced stability, precise drug loading, and controlled, triggered release [50,51,52]. Advanced designs incorporate multiple functionalities by combining targeting ligands, such as folate and peptides, with microenvironment-responsive motifs, thereby enabling site-specific accumulation and release [39,53,54]. Additionally, the co-delivery of therapeutic agents, including siRNA targeting programmed death ligand 1 (siPD-L1) and triptolide or other organelle-targeting moieties, facilitates synergistic therapy and improves efficacy [39,40,46,55].

**Figure 4:**
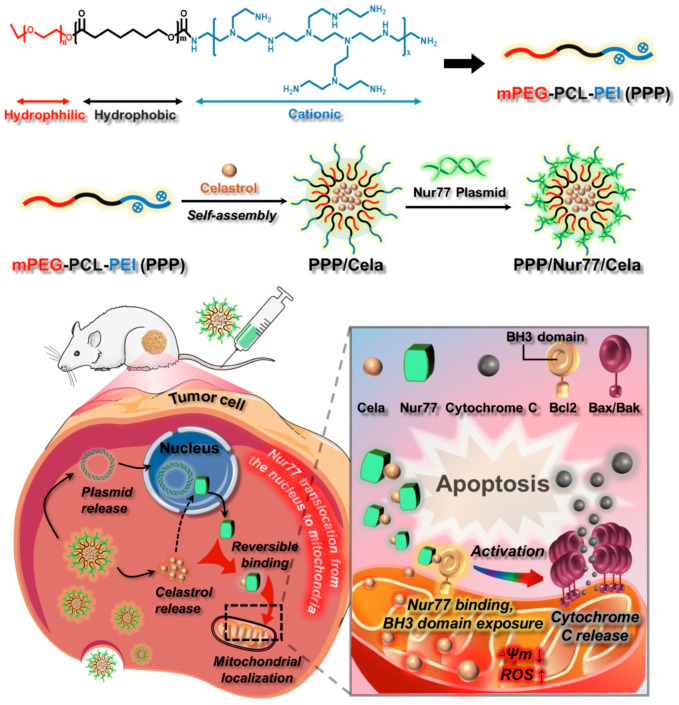
Schematic diagram showing preparation of celastrol micelles (PPP/Nur77/Cela co-delivery vehicle) and proposed mechanism of synergized chemo-gene therapy utilizing Cela to guide the gene-expressed Nur77 on targeted Bcl-2 protein conversion for cancer cell apoptosis developed by Liu et al. As a complementary strategy of combining chemotherapy and gene therapy, codelivery of Cela promoted Nur77 gene expression, directed Nur77 protein’s mitochondria relocation, increased drug sensitivity in drug resistance cancer cells, and activated mitochondrial dysfunction in Bcl-2 overexpression cancer cells. Reprinted with permission from reference [73]. Copyright 2023 Chemical Engineering Journal.

When surfactant concentration in water reaches a certain level, hydrophobic groups cluster together while hydrophilic groups face the water, forming micelles. Polymeric micelles self-assemble into nanospheres with a hydrophobic core and hydrophilic shell, enhancing drug protection and solubility [56]. Made from amphiphilic polymers, these micelles improve hydrophilicity, targeting, and environmental responsiveness [57,58,59,60,61,62,63]. They also accelerate chemical reactions, aiding in the study of biological processes. Capable of carrying various therapeutics, polymeric micelles are being tested in clinical trials for anti-cancer drug delivery. The design of celastrol-loaded polymeric micelles is centered on the modular integration of functional components to effectively overcome specific biological barriers and exploit disease microenvironments. This approach has evolved from basic solubilization techniques to advanced targeted and combination therapies. A representative example of this design strategy is the use of block copolymers, which are linear copolymers formed by chemically linking two or more polymer segments with distinct properties. Examples include diblock copolymers such as poly(ethylene glycol)-*b*-poly(propylene sulfide) (PEG-PPS) and poly(ethylene glycol)-*b*-poly(*ε*-caprolactone) (PEG-PCL), as well as triblock copolymers like poly(ethylene glycol)-poly(*ε*-caprolactone)-*g*-poly(ethylene imine) (PEG-PCL-PEI). These copolymers are characterized by their controllable molecular weight and customizable structure. This foundational strategy incorporates stimuli-responsive blocks to facilitate triggered drug release at pathological sites. For instance, ROS-sensitive linkages like poly(propylene sulfide) (PPS) and thioketal are designed to target inflamed or tumor tissues, reduction-sensitive disulfide bonds respond to elevated intracellular glutathione levels in tumor cells, and pH-sensitive boronic ester bonds enable drug release in acidic microenvironments [64,65,66,67,68,69,70]. Targeting efficacy is augmented through the use of surface ligands, such as folate or hyaluronic acid (HA), facilitating receptor-mediated uptake. Advanced systems further employ cascade targeting strategies [71,72]. Beyond the delivery of single agents like celastrol, micelles function as platforms for combination therapies, as demonstrated by the co-delivery of Nur77 to achieve synergistic chemo-gene therapy against resistant cancer (Figure 4), or through the surface conjugation of antigens for immunotherapy [73,74]. Additionally, the integration of micelles with biomaterials, such as thermosensitive hydrogels, enables the creation of injectable depots for sustained local drug release, which is particularly effective in treating ocular fibrotic diseases [75,76,77]. This progression from passive carriers to multifunctional, problem-solving nanosystems underscores the rational design of micelles tailored to address specific therapeutic challenges.

**Figure 5:**
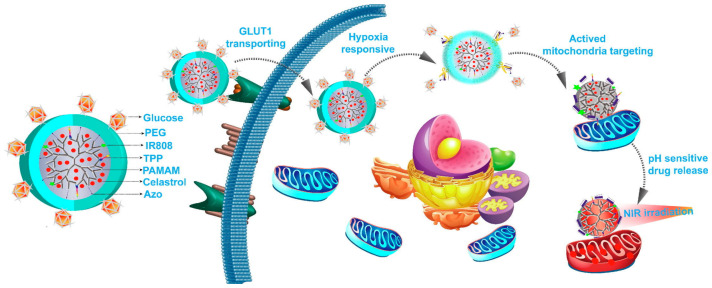
Schematic illustration of design principle and action mechanism about inhibiting tumor growth and metastasis of celastrol dendrimers (Glu-PEG-Azo/Mito-Cel808 complex) developed by Wei et al. The complex was actively transported by glucose transporter 1 (GLUT1)-overexpressing tumor cells. Subsequently, mitochondrial targeting was activated when PEG detached from PAMAM stimulated by the hypoxic tumor microenvironment. Finally, tumor mitochondria were completely destroyed after Cel were rapidly released in the alkaline mitochondrial matrix and 1-(5-carboxypentyl)-2-[2-[3-[[1-(5-carboxypentyl)-1,3-dihydro-3,3-dimethyl-2*H*-indol-2-ylidene]ethylidene]-2-chloro-1-cyclohexen-1-yl]ethenyl]-3,3-dimethyl-3*H*-indolium bromide (IR808) produced great heat with laser irradiation. Thus, the complex possessed superior specificity and efficiency in inhibiting tumor growth and metastasis. Reprinted with permission from reference [81]. Copyright 2022 Journal of Colloid and Interface Science.

Dendrimers are highly controlled, hyperbranched polymers with complex three-dimensional structures. Their exterior functional groups allow for biomolecule or contrast agent attachment, while drugs can be loaded inside. They are mainly studied for delivering nucleic acids and small molecules, often using charged polymers like poly(ethylenimine) (PEI) and poly(amidoamine) (PAMAM). Some dendrimer-based products are in clinical trials for use as theranostic agents, transfection agents, topical gels, and contrast agents [78]. Dendrimer-based nanosystems for the delivery of celastrol have been developed to address the dual challenge of achieving specific accumulation and subcellular targeting. This is primarily accomplished through the surface engineering of dendrimers. One approach involves the conjugation of targeting ligands, such as epithelial cell adhesion molecule (EpCAM) aptamers, to ensure highly specific recognition and enhanced tumor accumulation, as demonstrated in colon cancer models [79,80]. Comparative analyses within this framework indicate that aptamer-based conjugates may provide superior tumor penetration and efficacy compared to their antibody-based counterparts. In addition to cellular targeting, a more advanced strategy emphasizes organelle-specific delivery, particularly to mitochondria, by incorporating stimuli-responsive elements. For instance, a system that responds to the hypoxic tumor microenvironment by removing a PEG cloak to activate mitochondrial targeting enables site-specific chemothermal combination therapy (Figure 5) [81]. These designs exemplify the evolution from simple ligand–receptor targeting to sophisticated, conditionally activated systems for precise intracellular drug delivery.

Overall, polymeric nanoparticles represent an optimal choice for drug delivery applications due to their biodegradability, water solubility, biocompatibility, biomimetic properties, and stability during storage. The ease with which their surfaces can be modified enhances their capacity for targeted delivery, facilitating the transport of drugs, proteins, and genetic materials to specific tissues. This capability renders them particularly valuable in the fields of oncology, gene therapy, and diagnostics. In polymeric nanoparticles, natural polymers and synthetic polymers offer distinct advantages and limitations, while drug loading via physical encapsulation versus chemical conjugation further dictates performance outcomes. Natural polymers generally provide superior biocompatibility, biodegradability, and low immunogenicity, making them attractive for in vivo applications, but they often suffer from batch-to-batch variability, mechanical weakness, and potential enzymatic degradation. Synthetic polymers, conversely, offer precise tunability of molecular weight, degradation rate, and mechanical properties, enabling reproducible nanoparticle formulations with controlled release profiles, yet they may elicit inflammatory responses or require organic solvents during preparation. When comparing physical encapsulation (where the therapeutic is entrapped within the polymer matrix via hydrophobic, electrostatic, or hydrogen bonding interactions) to chemical conjugation (where the drug is covalently linked to the polymer backbone), head-to-head outcomes reveal clear trade-offs. Physical encapsulation typically achieves higher initial drug loading capacity and simplicity of formulation, but often leads to burst release and incomplete release profiles due to diffusion-driven leakage. Chemical conjugation provides enhanced stability in circulation, reduced premature release, and more tunable release kinetics via hydrolytically or enzymatically cleavable linkers, albeit often at the cost of lower overall loading efficiency, complex synthesis, and potential bioactivity loss of the conjugated drug. In therapeutic efficacy studies, conjugation strategies generally outperform encapsulation for targeted and sustained delivery by minimizing off-target effects and extending half-life, whereas encapsulation may be preferred for rapid, high-dose local delivery or when the drug lacks suitable conjugation handles. Ultimately, the optimal choice depends on the clinical context: natural polymers with encapsulation suit short-term, biocompatible delivery, while synthetic polymers with conjugation excel in long-circulating, controlled-release nanomedicines. Nonetheless, the application of polymeric nanoparticles is not without challenges, as they are associated with an elevated risk of particle aggregation and potential toxicity. Presently, only a limited number of polymeric nanomedicines have received approval from the Food and Drug Administration (FDA) and are in clinical use. However, numerous polymeric nanocarriers are currently undergoing evaluation in a variety of clinical trials.

#### 2.1.2. Lipid Nanoparticles

Lipid nanoparticles are usually spherical structures with lipid bilayers encasing internal water compartments. They are advantageous as delivery systems due to their simple formulation, self-assembly, biocompatibility, high bioavailability, capacity for large payloads, and adjustable physicochemical properties. These nanoparticles form micellar structures within their core, which can be modified through formulation and synthesis adjustments. Lipid nanoparticles consist of four main components: cationic or ionizable lipids for genetic material binding and endosomal escape, phospholipids for structure, cholesterol for stability and fusion, and PEGylated lipids for enhanced stability and circulation. Their easy synthesis, compact size, serum stability, and effective delivery make them crucial for personalized genetic therapies. Ionizable lipid nanoparticles are particularly suitable for delivering celastrol, as they maintain a near-neutral charge under physiological conditions but become charged in acidic environments, facilitating endosomal escape for intracellular delivery [82,83,84,85,86,87]. Hybrid and biomimetic nanoparticles overcome the limitations of single-material carriers by integrating complementary properties to enhance drug delivery. Their design is primarily driven by the need for improved targeting, controlled release, and immune evasion. A foundational strategy involves the use of lipid-based cores, such as phospholipid complexes and liquid crystalline nanoparticles, to increase drug solubility and facilitate passive accumulation [88,89]. Progress in the field is marked by the development of lipid-polymer hybrids, which merge the high drug-loading capacity of polymers with the biocompatibility of lipids. These hybrids can be further engineered with pH-responsive release mechanisms or functional surfaces, such as selenium, to improve oral bioavailability and exert anti-inflammatory effects [90,91]. The most advanced strategy entails biomimetic cloaking, wherein nanoparticles are enveloped with natural cell membranes, such as those derived from cancer cells or leukocytes [92,93]. This “camouflage” exploits the intrinsic homing capabilities and immune compatibility of source cells, facilitating targeted delivery to tumors or inflamed joints while minimizing systemic clearance, thus ensuring precise delivery and reduced toxicity. Nonetheless, despite these benefits, lipid nanoparticles may still face limitations due to low drug loading capacity and biodistribution challenges, which can lead to significant uptake by the liver and spleen.

#### 2.1.3. Protein Nanoparticles

**Figure 6:**
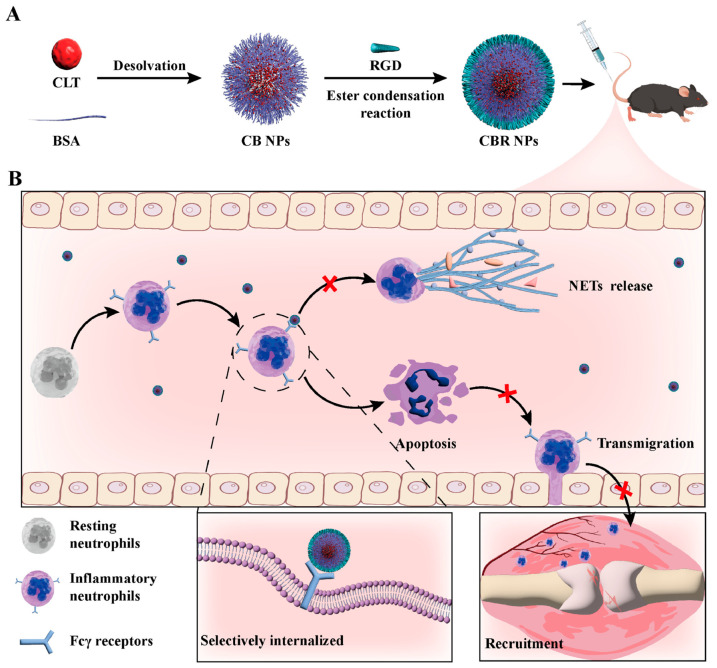
Schematic illustration of celastrol (CLT)-bovine serum albumin (BSA) nanoparticles (CBR NPs) as a treatment for rheumatoid arthritis developed by Liu et al. (**A**) Preparation of CBR NPs. (**B**) After intravenous injection, CBR NPs were selectively internalized by inflammatory neutrophils (INEs), and CLT released from CBR NPs promoted the apoptosis of INEs and inhibit the release of neutrophil extracellular traps (NETs). Reprinted with permission from reference [98]. Copyright 2024 Acta Biomaterialia.

Protein nanoparticles are highly structured entities formed by the assembly of protein molecules, employing the systematic evolution of engineered proteins and integrating the chemical properties of small molecules [94]. In comparison to other biomaterials, proteins exhibit superior permeability and hepatic uptake characteristics [95]. Protein-based nanocarriers, particularly albumin, leverage endogenous transport pathways for targeted delivery, with modifications facilitating tissue-specific applications. Native albumin nanoparticles exploit the intrinsic role of protein in nutrient transport and receptor-mediated transcytosis, thereby enhancing systemic drug delivery and improving pharmacokinetics, as evidenced in obesity treatment [96]. Targeted delivery is achieved by functionalizing albumin with specific ligands: lactosylation directs nanoparticles to hepatocytes via asialoglycoprotein receptors for liver diseases; RGD peptides target inflammatory neutrophils for rheumatoid arthritis (Figure 6); and brain-targeting peptides enable the penetration of blood–brain barrier for glioma therapy [97,98,99]. In addition to albumin, other proteins such as lactoferrin offer alternative targeting capabilities. Their hydrophilic backbones and functional groups facilitate the chemical conjugation of hydrophobic drugs, resulting in stable, high-loading nanoconjugates for synergistic cancer therapy [100]. This shift from passive and endogenous transport to active, ligand-directed targeting underscores the versatility of protein platforms in achieving organ- and disease-specific delivery of celastrol. Despite the advantages associated with protein nanoparticles in drug delivery, several challenges persist. The preparation process is influenced by a variety of factors, including the source, purity, and environmental conditions of protein, which can lead to batch variability and impact their stability in clinical applications. Furthermore, the size and shape of these nanoparticles may change in vivo, affecting their distribution and targeting efficacy. Additionally, their degradation rate is contingent upon varying physiological conditions, necessitating further research to optimize their behavior within the body.

#### 2.1.4. Inorganic Nanoparticles

**Figure 7:**
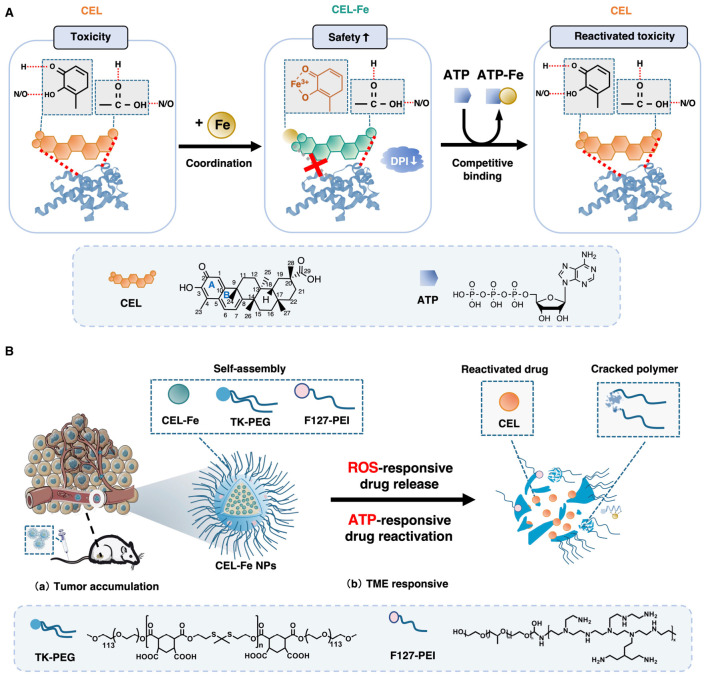
Schematic illustration of intelligent celastrol-ferrum nanoparticles (CEL-Fe NPs) designed by Li et al. (**A**) Formation and dissociation of CEL-Fe chelate which was proposed as a toxicity reduction strategy based on coordination bonds according to the corresponding structure-activity relationships. The reduction in hydrogen bonds led to a decrease in the binding affinity of CEL-Fe for its target proteins, resulting in reduced toxicity. ATP was used as a competitive binding agent to Fe(III). The antitumor efficacy of CEL was activated when coordination bonds between CEL and Fe(III) were broken. (**B**) Formation of CEL-Fe NPs and their responsiveness to ATP and ROS at the tumor site. This NP formulation enabled tumor accumulation through the EPR effect and led to drug release and drug activation triggered by ROS and ATP, respectively. Reprinted with permission from reference [104]. Copyright 2024 Science Advances.

**Figure 8:**
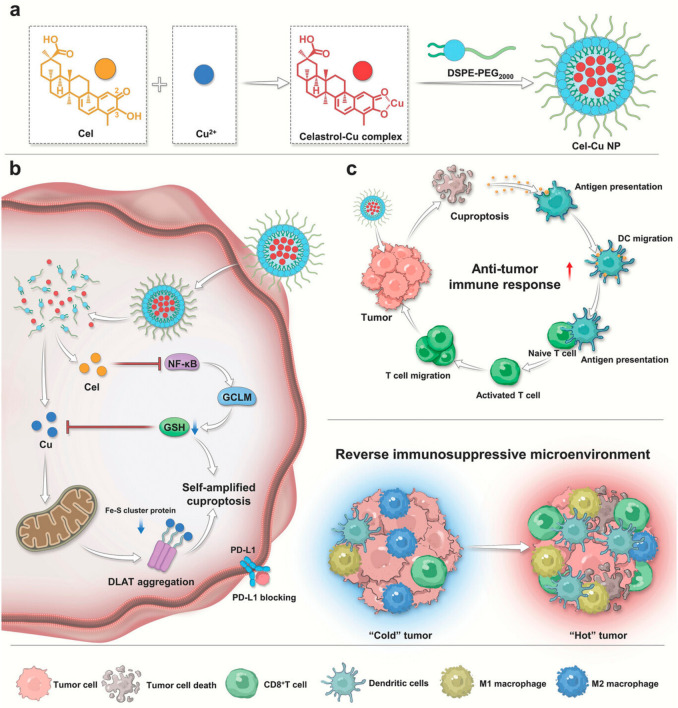
Schematic illustration of celastrol-copper nanoparticles (Cel-Cu NP) for self-amplified cuproptosis developed by Lu et al. (**a**) Preparation of Cel-Cu NP. (**b**) Biological mechanism of self-amplified cuproptosis induced by Cel-Cu NP, which simultaneously released Cu and Cel in tumor cells upon cell internalization. The released Cu bound to lipoylated dihydrolipoamide *S*-acetyltransferase (DLAT), hence triggering cuproptosis. Meanwhile, the released Cel inhibited the nuclear factor kappa-B (NF-κB) pathway to scavenge glutathione (GSH) in tumor cells, which enhanced the efficacy of cuproptosis in a self-amplified manner. (**c**) The self-amplified cuproptosis further induced immunogenic cell death (ICD) to reverse immunosuppressive tumor microenvironment, which augmented cancer immunotherapy. Reprinted with permission from reference [105]. Copyright 2024 Advanced Materials.

Inorganic materials like gold, iron, and silica are utilized to create nanostructured materials for drug delivery and imaging, offering customizable sizes and shapes. Their unique magnetic, radioactive, or plasmonic properties make them ideal for diagnostics, imaging, and photothermal therapies, providing biocompatibility and stability for niche applications beyond the capabilities of organic materials [101,102,103]. Inorganic and coordination-based nanocarriers harness distinct physicochemical characteristics to develop advanced delivery systems, exemplified by stimuli-responsive degradation, high porosity, and metal-drug synergism. Coordination methodologies employ metal ions to form complexes with celastrol, either to generate prodrugs activated by specific tumor metabolites such as adenosine triphosphate (ATP) (Figure 7) or to exploit the inherent biological functions of metals, as demonstrated in copper-coordinated nanoparticles that induce cuproptosis while enhancing immune responses (Figure 8) [104,105]. Porous frameworks, particularly metal–organic frameworks (MOFs) such as zeolitic imidazolate framework-8 (ZIF-8), exhibit the capacity for substantial drug loading within their crystalline matrices and undergo dissociation within the acidic tumor microenvironment, facilitating controlled drug release [106,107,108]. Similarly, mesoporous silica nanoparticles serve as robust scaffolds for substantial drug payloads, with surfaces amenable to functionalization using pH-responsive capping agents like chitosan, thereby enabling targeted release in tumor sites or inflamed joints [109,110]. A significant advantage of these inorganic platforms lies in their ability to co-deliver functional components, such as calcium carbonate, to buffer tumor acidity and enhance immunotherapy, or mitochondrial-targeting peptides to sensitize resistant cancer cells [110,111]. This strategy effectively integrates celastrol into multifunctional, metal- or mineral-enhanced combination therapies. Nonetheless, their clinical application is constrained by issues of low solubility and potential toxicity, particularly in formulations incorporating heavy metals.

Overall, nanoparticles have been extensively utilized across various disciplines (Table 1 and Table 2). In recent years, celastrol nanoparticles, encompassing both organic and inorganic types, have predominantly been employed in the treatment of tumors affecting digestive, reproductive, nervous, respiratory, and urinary systems. Specifically, within the realm of organic nanoparticles where celastrol serves as the active ingredient, polymeric nanoparticles have emerged more frequently as significant agents in anti-tumor therapy, whereas lipid nanoparticles have demonstrated greater therapeutic efficacy in the management of inflammatory conditions. Polymeric nanoparticles have predominantly been employed to enhance the anti-tumor efficacy of celastrol, with intravenous injection remaining the most prevalent route to achieve optimal bioavailability. In comparison to other nanocarriers, lipid nanoparticles have emerged as the most representative carriers for improving celastrol delivery, addressing challenges related to poor absorption and limited oral bioavailability in the treatment of inflammation. The high affinity of lipid materials for cell membranes, which effectively augments transmembrane influx and facilitates caveolae/clathrin-mediated endocytosis, is believed to enhance the absorption of celastrol within lipid nanoparticles. Additionally, the preferential lymphatic transport of lipid nanocarriers, which allows them to circumvent hepatic metabolism, constitutes a further advantage for improved absorption.

When preparing nanoparticles, focus on selecting materials with ligand modifications and porous structures. Optimizing the preparation process can enhance drug loading. Approved nanomaterials with good biocompatibility can mitigate safety concerns. Identifying specific receptors and ligands can reduce toxicity by limiting distribution to non-target tissues. Nanoparticles can enhance cargo stability, solubility, membrane transport, and circulation time, boosting safety and efficacy. Consequently, nanoparticle-related research is extensive and shows promise in small animal models. Nanoparticles serve as carriers for celastrol in diagnostics and therapy, but their clinical use hinges on factors like physical and chemical properties, drug loading and release efficiency, and carrier toxicity. While promising, some studies show nanoparticles can accumulate in cells and cause organ-specific toxicity, highlighting the urgent need for safer designs and stringent toxicity testing guidelines. When nanoparticles contact organisms, they quickly form a protein corona on their surface, altering their biological characteristics. This corona is the natural state of nanoparticles in vivo, unlike in vitro. The composition of protein corona is influenced by the size, shape, and surface modification of nanoparticles, as well as environmental factors like medium composition, incubation time, temperature, and pH. The reticuloendothelial system eventually clears the corona. It affects nanoparticle interactions with organisms, impacting uptake, distribution, and toxicity. However, the specific binding of protein molecules to nanoparticles and methods to track these changes in living organisms remain unclear and need future research.

### 2.2. Vesicles

**Figure 9:**
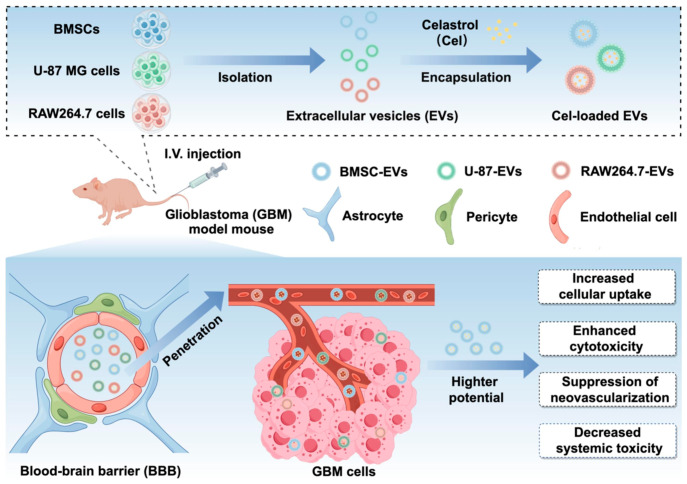
Schematic representation of preparation of celastrol-loaded extracellular vesicles (Cel-loaded EVs) derived from different sources and spatiotemporally sequential drug delivery of payload from blood–brain barrier (BBB)-crossing to glioblastoma cells developed by Zhang et al. Through the comparison of drug-loading capacity, intracellular delivery, cytotoxicity against glioblastoma cells and in vivo biodistribution, the selected EVs, as superior natural nanovehicles, were expected to exert better chemotherapy effect and decrease systemic toxicity of Cel. Here, higher EV production and Cel entrapment efficiency were observed in BMSC-EVs, and sequential delivery from BBB to glioblastoma cells was facilitated by BMSC-EVs, which significantly suppressed glioblastoma growth and increased biosafety. Reprinted with permission from reference [143]. Copyright 2024 International Journal of Pharmaceutics.

Amphiphilic molecules can form vesicles in water, which are colloidal bilayer structures that enclose aqueous compartments. These vesicles are crucial for storing, transporting, and digesting products in cells due to their low immunogenicity and high biocompatibility. In drug discovery and delivery, vesicles can carry both hydrophilic and hydrophobic drugs with minimal toxicity, extended drug action, and controlled release. They can be chemically modified to evade immune system, enhance drug solubility, and improve release in acidic environments. Additionally, vesicles can be linked with antibodies or aptamers to target specific receptors [119,120,121,122,123,124,125,126,127,128,129,130,131,132,133,134,135,136,137,138,139,140,141,142]. As biomimetic nanocarriers derived from cell membranes, vesicles leverage the inherent biological properties of their source cells to facilitate targeted delivery and enhance biocompatibility. The central scientific inquiry pertains to how the cellular origin of vesicles influences their intrinsic targeting tropism, production yield, and therapeutic efficacy. For example, extracellular vesicles sourced from bone marrow mesenchymal stem cells demonstrate innate tumor-homing abilities, rendering them particularly effective for targeting glioblastoma, with enhanced cellular uptake and diminished systemic toxicity compared to unencapsulated drugs (Figure 9) [143]. Furthermore, vesicles derived from erythrocyte membranes, which capitalize on the prolonged circulation and immune evasion characteristics of their parent cells, are engineered for sustained systemic delivery and preferential liver accumulation, thereby modulating macrophage polarization to ameliorate drug-induced liver injury [144]. This comparison underscores a design principle wherein the therapeutic goal directly informs the optimal selection of vesicle source, utilizing natural delivery mechanisms for precision medicine, whether through active tumor targeting or passive systemic delivery to a filtering organ.

#### 2.2.1. Liposomes

**Figure 10:**
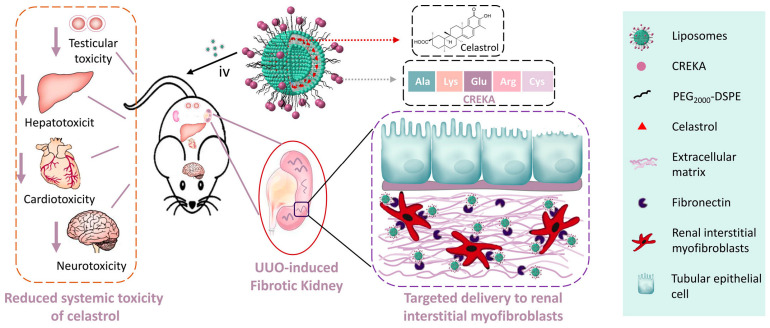
Targeted delivery of celastrol (CEL) to renal interstitial myofibroblasts using CREKA-coupled liposomes (CREKA-Lip) attenuated renal fibrosis and reduced systemic toxicity developed by Li et al. CREKA-Lip were specifically accumulated in renal interstitial myofibroblasts, and CEL-loaded CREKA-Lip, as a promising universal and safe fibrotic kidney carrier, showed enhanced antifibrotic effects and significantly decreased systemic toxicity induced by free CEL. Reprinted with permission from reference [153]. Copyright 2020 Journal of Controlled Release.

Liposomes, composed of lipid molecules, encapsulate hydrophobic drugs to enhance water solubility, bioavailability, and reduce side effects. They can be modified with phospholipids, cholesterol or sodium deoxycholate, and surface peptides to improve drug absorption and decrease toxicity. Liposomes form unilamellar and multilamellar structures, allowing them to carry both hydrophobic and hydrophilic drugs. Their stability and delivery efficiency can be adjusted by altering size, surface charge, lipid composition, and surface modifications. Often modified to avoid rapid uptake by reticuloendothelial system, liposomes are widely used in clinical settings and are the most common FDA-approved nanomedicines [145,146,147,148,149]. The engineering of liposomal delivery systems for celastrol aims to address its pharmacokinetic challenges and facilitate targeted therapy. This has led to the evolution of design strategies from simple formulations to advanced multifunctional systems. Traditional liposomes primarily function to enhance drug solubility, extend circulation time, and enable passive tumor targeting through enhanced permeability and retention (EPR) effect, as evidenced in their application for treating tumors, inflammation, and viral infectious diseases [150,151,152]. To achieve active targeting, liposomes are modified on their surface with ligands such as peptides (CREKA, Figure 10), saccharides (mannose and galactose), and glycosides (alkyl glucoside), which specifically bind to receptors on targeted cell types [153,154,155,156,157]. Advanced designs in drug delivery systems now incorporate stimuli-responsive and cascade-targeting mechanisms. An illustrative example is a HA-coated liposome that undergoes charge reversal upon degradation by lysosomal enzymes, subsequently revealing a mitochondrial-targeting moiety for precise subcellular delivery [158]. In the context of oral administration aimed at colonic targeting, polysaccharide coatings such as pectin and chitosan facilitate pH-dependent release and mucoadhesion [159]. Moreover, liposomes are increasingly utilized as platforms for synergistic combination therapies, co-delivering agents that either modulate the tumor microenvironment (tetrandrine) or sensitize tumor cells (copper complexes), thereby enhancing the therapeutic efficacy of celastrol [160,161]. This evolution underscores the rational design of liposomes, transitioning from merely improving bioavailability to enabling sophisticated and multimodal therapeutic strategies.

#### 2.2.2. Exosomes

Exosomes are small discoid vesicles comprising complex RNA and proteins, predominantly originating from multivesicular bodies formed through lysosomal invagination within cells. These vesicles subsequently fuse with cell membranes to release their contents into extracellular matrix [162,163,164,165,166,167,168,169,170,171,172,173,174,175]. Exosome-based delivery systems are engineered to exploit inherent homing capabilities and biocompatibility of extracellular vesicles for targeted therapeutic applications, with functionalization strategies playing a crucial role in determining their specificity. A fundamental design principle involves the surface modification of exosomes to facilitate active targeting. This is illustrated by the conjugation of folic acid to macrophage-derived exosomes, which enables the precise delivery of celastrol and resveratrol to folate receptor-positive cells, such as activated macrophages, for synergistic anti-inflammatory therapy [176]. Beyond targeting cell surface, a more sophisticated approach involves directing therapeutic agents to specific subcellular organelles. This is accomplished by modifying the surface with targeted peptide signals, such as KDEL, which guide vesicles to endoplasmic reticulum. This strategy not only enhances drug accumulation at this organelle, thereby inducing immunogenic cell death, but also facilitates the co-delivery of siRNA to inhibit programmed cell death ligand 1 (PD-L1), resulting in a potent combinatorial immunotherapy for cancer [177]. These instances exemplify the evolution from employing exosomes as basic, naturally targeted carriers to engineering them as precision instruments for both cellular and subcellular delivery. Exosomes have been studied as drug delivery carriers in recent years due to their nanosize. While exosomes have been obtained from biological fluids and cell culture media, these methods are highly inefficient.

#### 2.2.3. Phytosomes

Phytosomes represent stable complexes resulting from charge transfer interactions between pharmaceutical agents and phospholipids, thereby altering physicochemical properties and enhancing bioavailability through straightforward and cost-effective preparation methods [178,179]. Nanocarriers based on phytosomes leverage the natural affinity between celastrol and phospholipids to construct stable lipid-bilayer configurations, which significantly improve the oral bioavailability and cellular uptake of this hydrophobic compound. A notable advancement within this platform is the incorporation of functional elements, such as selenium, to develop synergistic and multifunctional systems. The selenium-deposited celastrol phytosome fulfills a dual function: the phytosome structure enhances transepithelial transport and improves drug release, while the incorporated selenium serves as an anti-inflammatory sensitizer. This combination yields a nanomedicine that not only facilitates more effective delivery of celastrol but also co-delivers a therapeutic mineral, thereby enabling a dual-action mechanism. The therapeutic outcome is a robust inhibition of specific inflammatory pathways, particularly the suppression of NLRP3 inflammasome and associated pyroptosis in macrophages, which demonstrates efficacy in models of arthritis and diabetic nephropathy [180,181,182]. This design exemplifies the strategy of augmenting a natural drug–phospholipid complex with an active inorganic component to create a synergistic, disease-modulating nanotherapy. Until now, phytosomes have hitherto been widely employed to improve the oral absorption of herbal extracts and increase the bioavailability of various plant-derived compounds, which were rarely reported in celastrol delivery-related research in the past five years.

#### 2.2.4. Niosomes

Niosomes, akin to liposomes, serve as vesicular carriers capable of encapsulating both hydrophilic and lipophilic drugs [183,184,185]. These structures are synthesized using nonionic surfactants, thereby eliminating the need for thermo-labile phospholipids and enhancing their stability and transdermal delivery capabilities. Niosome-based topical delivery systems are specifically engineered to improve the penetration and localized retention of celastrol within the skin, offering a promising therapeutic approach for dermatological conditions such as psoriasis. This formulation effectively addresses the significant challenge of delivering hydrophobic drugs into skin layers. When celastrol-loaded niosomes are incorporated into a hydrogel, they act as a sustained-release depot, accumulating within the skin upon topical application. This localized delivery method ensures a high concentration of celastrol at the site of pathology while minimizing systemic exposure, as demonstrated by the reduction in inflammatory markers in the bloodstream [186]. This strategy exemplifies a targeted topical nanomedicine approach, confining potent immunomodulatory effects of celastrol to affected tissues. Even though there are currently few relevant studies available, niosomes are still considered one of the best alternatives to liposomes due to their inherent enhanced skin penetration, greater chemical stability, and lower costs.

In general, liposomes are predominantly utilized for the delivery of celastrol within vesicular delivery systems, particularly in studies related to tumors and inflammation, akin to the application of nanoparticles (Table 3 and Table 4). Alternative dosage forms, such as exosomes, phytosomes, and niosomes, remain in the early stages of development and are less frequently incorporated into celastrol-containing formulations. Their preparation is often time-consuming, costly, and yields low output, rendering them unsuitable for large-scale production. To date, innovative dosage forms derived from traditional liposomes, including transfersomes, ethosomes, transethosomes, and invasomes, have been infrequently employed for celastrol delivery, warranting further exploration and application.

### 2.3. Emulsions

**Figure 11:**
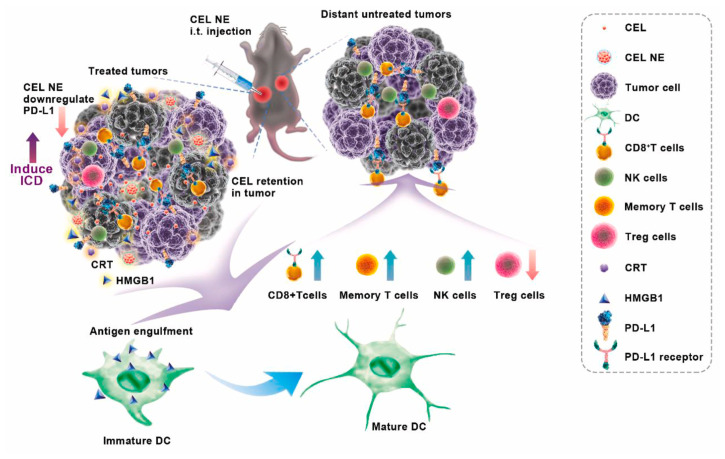
Schematic illustration of intratumorally (i.t.) injected celastrol nanoemulsion (CEL NE) simultaneously inducing ICD and programmed cell death-ligand 1 (PD-L1) downregulation and boosting systemic abscopal effects on B16F10 bilateral tumor model developed by Qiu et al. CEL NE intrathecally injected in the subcutaneous tumor on one side continuously released CEL and induced tumor cells to expose calreticulin (CRT) and release high mobility group box 1 (HMGB1) as tumor-associated antigens, which were engulfed by antigen-presenting cells (dendritic cells, DCs) and primed CD8^+^ T cell infiltration and activation. Meanwhile, CEL NE also effectively downregulated PD-L1 expression in tumor cells. The synergy of strong ICD and PD-L1 reduction activated the tumor immunosuppressive microenvironment and effector CD8^+^ T cells, giving potent tumor inhibition of both primary tumor and distant contralateral tumor as well as long-lasting systemic tumor suppression. Reprinted with permission from reference [203]. Copyright 2021 Biomaterials.

Emulsions are stable mixtures of oil and water with emulsifiers (surfactants) and co-emulsifiers (co-surfactants), offering high targeting capabilities and reducing adverse effects [187,188,189,190,191]. Nanoemulsions, unlike microemulsions, have gained interests due to their dynamic stability and thermodynamic instability, requiring more energy to form but allowing for a broader and safer selection of surfactants [192,193,194,195,196,197,198,199,200,201,202]. Nanoemulsion-based formulations are engineered to convert celastrol into a systemically administrable or locally injectable agent capable of modulating specific disease microenvironments, with therapeutic objectives guiding design strategies. In the context of local immunotherapy, nanoemulsions injected intratumorally function as a depot, facilitating the sustained release of celastrol to induce immunogenic cell death and downregulate PD-L1 expression within tumor tissues. This localized mechanism, operating independently of checkpoint inhibitors, initiates a systemic antitumor immune response that can inhibit both primary and metastatic tumors, thereby effectively transforming localized chemotherapy into in situ vaccination (Figure 11) [203]. In contrast, for systemic delivery to diffuse inflammatory sites such as arthritic joints, intravenous nanoemulsions are meticulously engineered for active targeting. By modifying the surface of a biocompatible lipid emulsion core with dextran sulfate, the formulation is designed to achieve specific binding to scavenger receptors on synovial macrophages. This strategic design ensures that the pro-apoptotic effect of celastrol is concentrated on pathogenic immune cells within inflamed joints, thereby mitigating systemic toxicity while effectively rebalancing the local inflammatory environment [204]. These instances exemplify how nanoemulsion design, whether for local depot or systemic targeted delivery, is specifically tailored to harness the immunomodulatory or pro-apoptotic activity of celastrol within a defined pathological context (Table 5 and Table 6). Historically, nanoemulsions have been less prevalent than nanoparticles in research concerning celastrol delivery, which has predominantly focused on treatments for tumors and inflammation. Thermodynamically unstable nanoemulsions are susceptible to aggregation and flocculation, and the potential biological toxicity of non-natural emulsifiers warrants further investigation.

### 2.4. Carbon Dots

Carbon dots represent a category of zero-dimensional nanomaterials characterized by exceptional fluorescent properties, consisting of dispersed near-spherical carbon particles of minute dimensions [205,206,207,208]. Their large specific surface area and favorable biocompatibility render them highly suitable for drug delivery applications, offering significant therapeutic potential. In comparison to free drugs, carbon dot-based delivery systems exhibit enhanced solubility and efficacy, alongside reduced side effects. Carbon dot-based nanoplatforms effectively integrate the therapeutic properties of celastrol with the distinctive optical and catalytic characteristics of carbon nanomaterials, facilitating synergistic combination therapies. This design strategically addresses the challenge of merging chemotherapy with photothermal therapy within a singular, stable agent. In this configuration, celastrol is covalently bonded to carbon dots, which are subsequently linked to poly(dopamine) (PDA), a known photothermal agent. Near-infrared irradiation plays a dual role by generating localized heat for photothermal therapy and concurrently triggering the release of celastrol from the carrier through the cleavage of amide bonds. Additionally, carbon dots exhibit peroxidase-like activity, catalyzing the formation of cytotoxic hydroxyl radicals within the tumor microenvironment. This multifunctional design culminates in a triple-action therapeutic approach, encompassing chemical, photothermal, and catalytic therapies, all synergistically activated by a single light stimulus. This innovative strategy presents a potent approach against resistant tumors [209].

### 2.5. Inclusion Complexes

Inclusion complexes serve as a form of nanoscale drug delivery system achieved through molecular encapsulation, which significantly enhances stability, solubility, volatility, irritability, bioavailability, dissolution rate, objectionable odor, and adverse reactions [210]. Cyclodextrin inclusion complexes represent a well-established approach to augment the aqueous solubility of hydrophobic compounds such as celastrol, with their effectiveness dependent on the specific host-guest interactions between drug molecules and cyclodextrin derivatives. A critical scientific consideration involves selecting the appropriate cyclodextrin variant to optimize the stability of complex and improve therapeutic outcomes. Comparative analyses indicate that sulfobutylether-*β*-cyclodextrin forms a more efficacious complex with celastrol compared to 2-hydroxypropyl-*β*-cyclodextrin. This enhanced performance is attributed to the increased binding affinity and cavity compatibility provided by sulfobutyl ether substituents. Consequently, the resulting complex exhibits improved solubility and stability in physiological media, enhanced intestinal permeability, and increased cytotoxic efficacy against cancer cells [211]. This example highlights the importance of rational molecular design of excipients within a specific formulation class for optimizing the biopharmaceutical performance of drugs.

### 2.6. Others

**Figure 12:**
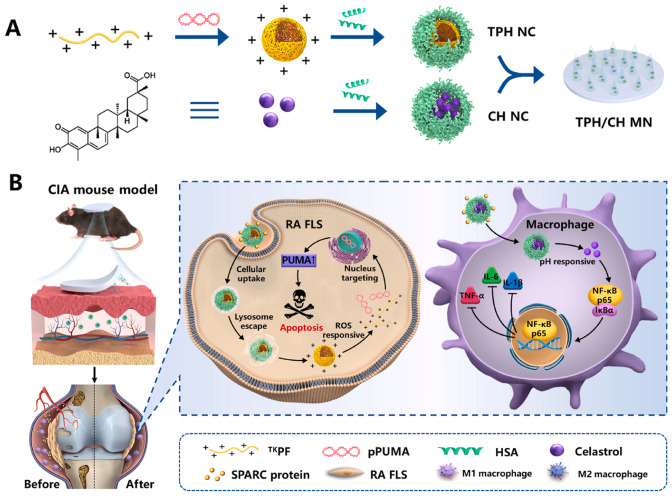
Schematic illustration of (**A**) fabrication procedures of HA-based celastrol (Cel) microneedles encapsulated with dual human serum albumin (HSA)-contained nanocomplexes (TPH/CH MN) and (**B**) rheumatoid arthritis therapeutic mechanism through inducing fibroblast-like synoviocyte (FLS) apoptosis and regulating inflammatory macrophages developed by Hua et al. A MN loaded with dual nanocomplexes was developed to realize both FLS apoptosis and inflammatory inhibition. Thioketal-crosslinked fluorinated polyethyleneimine 1.8 K (^TK^PF) exhibited higher transfection efficiency of p53 upregulated modulator of apoptosis (PUMA) plasmid in FLS. Cel@HSA nanocomplexes (CH NCs) with pH-responsive release property suppressed lipopolysaccharide (LPS)-stimulated inflammatory responses of RAW264.7 macrophages. TPH/CH MN attenuated collagen-induced arthritis symptoms, reduced inflammation infiltration, and relieved cartilage damage and bone erosion. Reprinted with permission from reference [219]. Copyright 2024 Bioactive Materials.

**Figure 13:**
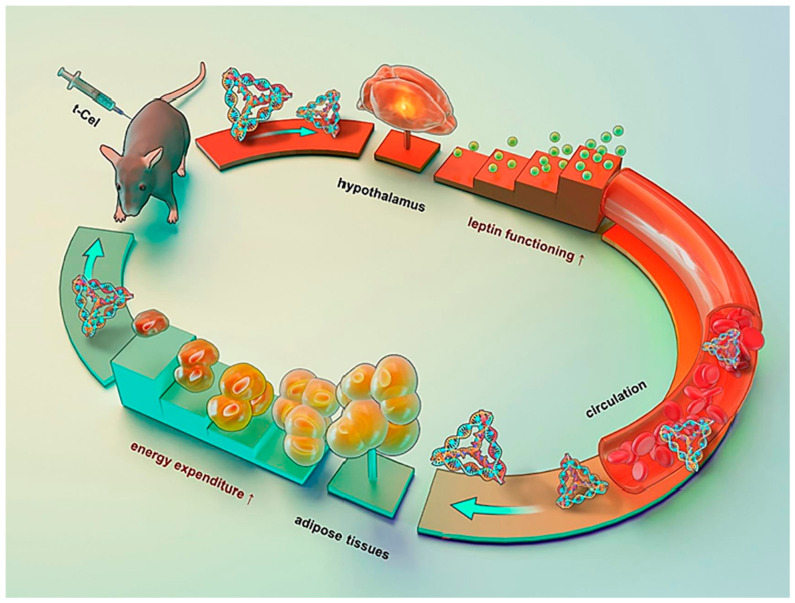
Celastrol-loaded tetrahedral framework nucleic acid (Cel-loaded tFNA, t-Cel), a “nano-patroller” simultaneously regulating hypothalamus leptin sensitivity and adipose energy expenditure designed by Liu et al. tFNA was used as an efficient delivery carrier for Cel for the first time, and t-Cel rescued leptin resistance, promoted energy expenditure, and inhibited obesity, inflammation, and insulin resistance. Reprinted with permission from reference [225]. Copyright 2024 Chemical Engineering Journal.

Advanced and emerging material platforms for celastrol delivery are being developed to exploit unique material properties, including magnetism, photothermal conversion, structural anisotropy, and biological mimicry. These platforms aim to facilitate novel therapeutic approaches or address specific challenges associated with drug administration, often by integrating multiple functionalities. For example, magnetite/hematite-based magnetic heterogeneous nanorods and ultrasound-mediated magnetic microbubbles not only deliver drugs but also incorporate imaging capabilities and can be controlled by external fields for triggered release [212,213]. Photothermal nanomaterials, such as graphene oxides, enable combined chemo-photothermal therapy, wherein near-infrared light induces drug release and exerts a synergistic ablative effect [214]. Structural materials, including nanoporous membranes and microspheres/microcapsules, are designed for controlled and sustained release in targeted anatomical locations [215,216,217,218]. Additionally, microneedle arrays facilitate transdermal delivery by bypassing the skin barrier, allowing for painless, localized administration of complex nanoformulations, such as gene-drug combinations, directly to specific sites (Figure 12) [219,220]. Biopolymer-based systems, such as chitosan conjugates, collagen implants, and hydrogel depots, enhance drug solubility, facilitate controlled release, and provide localized structural support [221,222,223,224]. Furthermore, biomimetic nanostructures, including tetrahedral framework nucleic acids (tFNA), represent an advanced frontier, leveraging programmable nucleic acid architectures for efficient cellular uptake and systemic delivery to modulate complex metabolic pathways (Figure 13) [225]. This diverse array of tools illustrates how material innovation is broadening the functional capabilities of celastrol formulations, extending beyond mere delivery to encompass theranostic and tissue-engineering applications (Table 7 and Table 8).

## 3. Barriers to Clinical Translation

Celastrol has yet to advance to a clinical trial for human use. Its long tenure as a purely research topic before the advent of nano-delivery is largely due to its paradoxical nature: it is a “treasure” of potent anti-inflammatory and anti-obesity activity that comes wrapped in the toxicity of its natural source. For decades, the clinical application of celastrol was stymied by severe physicochemical constraints. It is notoriously insoluble in water, has poor oral bioavailability, and exhibits dose-dependent toxicity that narrows its therapeutic window. Nanotechnology emerged not merely as an incremental improvement, but as an enabling strategy to overcome these fundamental barriers, allowing researchers to encapsulate celastrol to enhance its solubility, achieve targeted delivery, and mitigate systemic toxicity, thereby finally unlocking its translational potential. The current clinical development status of celastrol or celastrol-based formulations was briefly added to better connect the preclinical findings with potential clinical applications.

It is noteworthy that scaling up celastrol nanoformulations for clinical translation presents formidable regulatory and manufacturing hurdles. From a manufacturing standpoint, the inherent physicochemical properties of celastrol, such as poor aqueous solubility, high hydrophobicity, and conformational instability, necessitate complex, multi-step nanofabrication processes including nanoprecipitation, high-pressure homogenization, or microfluidics that are difficult to maintain with batch-to-batch consistency, particularly regarding particle size distribution, drug loading efficiency, and release kinetics, while also requiring stringent control over excipient quality and sterile processing, which drives up production costs and complicates scale-up from lab to pilot and commercial scales. On the regulatory front, these nanoformulations are typically classified as complex drug-device or novel biological hybrids, falling outside standard generic or small-molecule approval pathways; regulators demand extensive physicochemical characterization, robust in vitro-in vivo correlation (IVIVC) models, and rigorous toxicological assessments to address concerns over off-target accumulation, immunogenicity, and chronic toxicity, all of which are exacerbated by the lack of harmonized nano-specific guidance and reference standards. Moreover, the limited stability of celastrol in biological matrices and the potential for batch-dependent variations in protein corona formation further complicate the establishment of clinically meaningful specifications, necessitating adaptive, risk-based quality-by-design (QbD) approaches and close, early engagement with agencies like the FDA or European Medicines Agency (EMA) to navigate these intertwined challenges and avoid costly late-stage failures.

## 4. Discussion

The immense potential of celastrol has garnered significant attention in recent studies focusing on both its derivatives and drug delivery technologies [19,20,21,22]. The investigation and modification of naturally occurring scaffolds, informed by their bio-content and structural similarities, hold promise for improving the suboptimal characteristics of celastrol. Beyond structural modifications, advancements in drug delivery systems can enhance water solubility, increase bioavailability, improve target specificity, reduce toxicity, and enable the simultaneous delivery of diverse drugs. This approach offers a novel pathway for formulation development, quality control, and combined usage, which is anticipated to provide a sustainable, cost-effective, efficient, and environmentally friendly strategy for broadening the medicinal applications of celastrol. For example, the widespread application of celastrol across various therapeutic domains, due to its numerous targets, suggests its potential for the combination with multiple drugs. Combination therapy can be enhanced through drug delivery systems to achieve synergistic therapeutic effects of celastrol when used alongside other drugs, such as chemotherapeutic agents and immune checkpoint inhibitors, thereby realizing the objective of “1 + 1 > 2”. Multi-drug delivery systems facilitate the simultaneous delivery of agents for synergistic therapy, enhancing therapeutic outcomes by releasing different active ingredients at varying rates within the optimal time window. This approach increases bioavailability while reducing systemic toxic side effects. Consequently, the indications for celastrol can be expanded beyond tumors to include autoimmune diseases and metabolic disorders, addressing challenges such as chemotherapy resistance and immune resistance (Table 9).

### 4.1. Pharmacology and Toxicology

Celastrol exerts its broad pharmacological effects through a complex network of direct molecular targets and downstream signaling pathways. On a mechanistic level, celastrol functions as a covalent modifier, primarily targeting reactive cysteine residues on key proteins. Recent advances using ABPP have identified mitochondrial isocitrate dehydrogenases, including IDH2 and IDH3A, as direct molecular targets; its binding inhibits their activity, leading to metabolic reprogramming, the accumulation of oncometabolites, and ultimately the inactivation of the JAK/STAT pathway to induce ferroptosis and apoptosis in cancer cells. In parallel, celastrol exerts its well-documented anti-inflammatory and anti-proliferative effects by suppressing other pivotal signaling axes, notably the NF-κB pathway, and by inhibiting the JAK2/STAT3 pathway, which is critical for its anti-tumor activity in gastric and ovarian cancers. The pharmacokinetic/pharmacodynamic (PK/PD) relationship of celastrol is characterized by a significant disconnect between its potent activity and poor clinical usability, primarily due to extremely low oral bioavailability. This poor absorption is compounded by rapid systemic clearance and extensive tissue distribution, leading to a narrow therapeutic window where doses required for efficacy are perilously close to those causing severe systemic toxicity. The mechanisms underlying this systemic toxicity are directly linked to its off-target biodistribution. Following administration, free celastrol accumulates indiscriminately in major organs, with high concentrations found in the liver, heart, and brain. This non-specific distribution is the primary cause of its dose-limiting toxicities, which include significant hepatotoxicity (hepatocyte atrophy), cardiotoxicity (myofibrillar loss), and neurotoxicity (neuron pyknosis and liquefactive necrosis). This narrow therapeutic index and multi-organ toxicity profile are the principal barriers to its clinical translation, driving current research efforts toward advanced drug delivery systems, such as nanoparticle encapsulation and targeted liposomes, to improve its PK profile by enhancing target-site accumulation and minimizing off-tissue exposure.

### 4.2. Biosafety

The safety of nanocarriers remains a critical consideration for their clinical translation, particularly regarding immunotoxicity, long-term biodistribution, and chronic adverse effects. Immunotoxic responses, including complement activation, cytokine storms, and hypersensitivity reactions, are often triggered by the size, surface charge, and protein corona formation of nanocarrier, which can lead to off-target inflammation or immunosuppression. Regarding biodistribution, nanocarriers tend to accumulate predominantly in the liver and spleen due to the mononuclear phagocyte system, resulting in prolonged retention and potential organ-specific toxicity, such as hepatocyte necrosis, sinusoidal congestion, or splenic lymphoid follicle depletion. Chronic adverse effects may arise from persistent intracellular storage in Kupffer cells or dendritic cells, potentially leading to granuloma formation, oxidative stress, fibrosis, or disruption of iron homeostasis. Furthermore, slow degradation or non-biodegradable nanomaterials can cause lysosomal dysfunction and impair antigen presentation over extended periods, raising concerns about late-onset inflammation, autoimmunity, or carcinogenesis. Thus, thorough long-term in vivo studies assessing multiple dosing regimens and organ function over months to years are essential to establish safety thresholds and design biodegradable and low-immunogenic nanocarriers for chronic or repetitive administration. Within the realm of pharmaceutical research, the evaluation of the biological safety of celastrol preparations predominantly relies on basic cytotoxicity assessments. However, it is imperative to also monitor potential adverse reactions comprehensively. Many nanocarriers with suboptimal specific drug release performance may result in burst release, excessive local concentrations, and considerable toxicity. Consequently, enhancing sustained and controlled release properties, reducing the frequency of administration, maintaining appropriate plasma concentrations, and increasing therapeutic stability represent key areas for future research. To address the intrinsic issue of high toxicity and low biocompatibility of celastrol itself, targeting celastrol delivery deserves in-depth study, in which restricted accumulation in kidney, liver, spleen, and other off-targeted organs will result in improved therapeutic activities and reduced toxic side effects. Precise drug delivery to diseased cells or tissues can materialize via targeted delivery strategies including passive targeting utilizing EPR effects and active targeting achieved by ligand (peptides/antibodies/small molecules) modifications. Attention should be paid to the long-term toxicity and immunogenicity of nanocarriers themselves and celastrol-loaded delivery systems, as well as their impacts on gut microbiota.

### 4.3. Emerging Approaches

The evolution of nanomedicine will be increasingly defined by emerging approaches that move beyond traditional passive targeting and static drug delivery. Biomimetic carriers, such as cell membrane-coated nanoparticles and extracellular vesicle-mimetic systems, are poised to harness natural biological interactions to enhance circulation time, immune evasion, and tissue-specific homing. Concurrently, stimuli-responsive systems that exploit endogenous (pH, enzymes, and redox gradients) or exogenous (light, ultrasound, and magnetic fields) triggers will enable on-demand drug release with spatiotemporal precision, minimizing off-target effects. Most transformative, however, will be the integration of personalized nanomedicine strategies, wherein patient-specific genomic, proteomic, and immune profiles guide the design of nanocarriers from lipid nanoparticles tailored to individual tumor mutations to adaptive systems that recalibrate dosing in real time. The convergence of these approaches promises not only to improve therapeutic indices but also to establish a dynamic, feedback-driven platform for truly individualized treatment regimens.

In addition to their application in drug delivery, nanoplatforms demonstrate multifaceted regulatory capabilities concerning mechanisms of action. In the regulation of mitochondrial metabolic flux, nanocarriers mimic the structure of phospholipid bilayer of cell membranes, thereby enhancing the permeability of mitochondrial inner membranes and facilitating the transmembrane transport of key substances involved in energy metabolism. Regarding the modulation of endoplasmic reticulum stress responses, nanoparticles with surface modifications of serine or proline groups can specifically bind to endoplasmic reticulum stress sensor proteins, effectively terminating unfolded protein responses through physical blocking mechanisms. With respect to lysosomal functional repair, pH-responsive nanovesicles enhance the activity of enzymes involved in biodegradation by altering the pH of intracellular microenvironment, inducing conformational changes in lysosomal membrane proteins. Given the potential of celastrol for further exploration in therapeutic applications and undiscovered mechanisms, diverse delivery systems are instrumental in advancing the comprehensive investigation of spatiotemporal mechanisms of action of celastrol within specific organelles.

### 4.4. Challenges for Clinical Application

Given the aforementioned potential of delivery strategies in celastrol-based combination therapy and the verification of target/signaling pathways, it is imperative to conduct an in-depth evaluation of their bioactivity and biosafety. As traditional Chinese medicine continues to modernize, preclinical studies on celastrol offer valuable insights for new drug development. However, existing limitations in its clinical application highlight the necessity for advancements in mass production and quality control to facilitate drug development and application. Scaling the production of complex nanomedicines such as exosomes, biomimetic nanoparticles, and MOFs introduces profound challenges beyond those encountered for conventional liposomes or polymeric particles. Industrial scaling requires transitioning from laboratory-scale synthesis to pilot and commercial batches while preserving critical quality attributes. A hurdle magnified for exosomes due to their biological origin, donor cell variability, and sensitivity to shear stress, and for MOFs due to non-linear reaction kinetics and solvent effects in large reactors. Good Manufacturing Practice (GMP) compliance demands that even biologically derived exosomes be produced under stringent contamination controls, with validated cell banks, serum-free media to avoid adventitious agents, and robust downstream purification such as tangential flow filtration and size-exclusion chromatography, while biomimetic nanoparticles such as cell-membrane-coated particles must ensure consistent membrane coating fidelity and orientation. Batch-to-batch reproducibility is notoriously difficult for exosomes because subtle changes in cell culture conditions such as oxygen, glucose, and passage number alter cargo loading, size distribution, and surface marker expression; for MOFs, slight variations in precursor mixing rates or temperature can yield different crystallite morphologies and porosities, directly affecting drug loading and release. Stability profiling is equally demanding: exosomes require cryogenic storage or lyophilization with lyoprotectants to prevent aggregation and cargo degradation, whereas MOFs may hydrolytically degrade or collapse over time in aqueous buffers, necessitating specialized coatings or excipients. Quality control (QC) for these complex systems cannot rely solely on simple pharmacopoeial tests; exosome batches must be characterized for particle concentration (nanoparticle tracking analysis), identity (tetraspanin profiling via flow virometry or Western blot), purity (protein/DNA contaminant levels), and potency (functional assays in target cells). Biomimetic nanoparticles need assays confirming membrane protein integrity and orientation such as sodium dodecyl sulfate polyacrylamide gel electrophoresis (SDS-PAGE) and enzyme-linked immunosorbent assay (ELISA), while MOFs require X-ray diffraction for crystallinity, Brunauer-Emmett-Teller (BET) surface area analysis, and inductively coupled plasma mass spectrometry for metal content. Regulatory pathways are still maturing: exosome therapeutics face ambiguous classification and agencies like the FDA and EMA demand extensive comparability protocols post-process changes, with a heavy burden to demonstrate that endogenous nucleic acids or proteins are free from aberrant immunogenicity. Manufacturing costs reflect this complexity: exosome production under GMP can cost tens of thousands of dollars per dose due to ultracentrifugation steps and quality release assays; biomimetic nanoparticles require costly membrane extraction and synthetic lipid anchors; MOF synthesis often uses expensive organic linkers, high-pressure reactors, and solvent-exchange steps that challenge cost-effectiveness. Ultimately, bridging lab-scale innovation to affordable GMP manufacturing for these platforms will require process analytical technology (PAT) for real-time monitoring, single-use bioprocessing equipment, and harmonized regulatory guidelines that distinguish between raw material variability and process-related impurities.

In parallel, the development of animal models that more closely mimic clinical practice is advantageous for assessing the efficacy and safety of pharmaceutical formulations. Currently, small animals such as (nude) mice, rats, and rabbits are extensively utilized to evaluate the pharmacological activities and physiological toxicities of test compounds, owing to their short life cycles, rapid reproduction rates, and cost-effectiveness. Nonetheless, the relatively brief lifespan of these small animals poses challenges for assessing the long-term efficacy and safety of drugs. In contrast, larger animals, including pigs, dogs, and monkeys, exhibit greater physiological similarities to humans, rendering them invaluable for research related to disease mechanisms and drug screening. The extended lifespan of large animals allows for the observation of pharmacological effects under prolonged administration. For instance, in oncology-related studies, experiments involving large animals can elucidate whether test drugs induce tumor recurrence or metastasis and precipitate other chronic conditions, with patient-derived tumor xenografts providing more compelling evidence. In the interim, various drug delivery routes possess distinct advantages and disadvantages, and novel routes of administration warrant further investigation.

### 4.5. Interdisciplinary Research

Drawing on the interdisciplinary integration of computer science, materials science, chemistry, biology, and medicine, the fields of pharmacology and pharmacy have significantly advanced through the application of artificial intelligence (AI). AI, recognized as an innovative and effective approach, leverages machine learning algorithms to process complex datasets, thereby expediting the development of drug carriers, predicting pharmacokinetic parameters, enhancing design efficiency and accuracy, and offering extensive potential applications in drug delivery systems. In contrast to traditional drug delivery systems, which are often time-intensive and laborious to design, AI-driven solutions markedly enhance practical application efficiency. Presently, AI-enabled drug delivery systems have yet to be applied to celastrol, highlighting an urgent area for further exploration.

## 5. Conclusions and Perspectives

This review paper examines recent advancements in the development of delivery strategies aimed at enhancing the efficient administration of celastrol for disease treatment. By detailing the design principles, fabrication methods, and efficacy–safety assessments of these systems, we aim to offer a comprehensive analysis and underscore their significance. The rational and innovative design of celastrol delivery systems presents promising prospects for its application in clinical settings as a localized, responsive, and personalized therapeutic option for patients.

## Figures and Tables

**Table 1:** Summary of recent celastrol-related studies on nanoparticle formulations.

Dosage Forms	Preparative Techniques	Composition	Encapsulation Efficiency	Loading Capacity	Particle Size	[Ref]
nanoparticles	self-assembly	NS	NS	NS	S-Cel NPs: 56.0 ± 19.7 nm	[27]
					E-Cel NPs: 125.1 ± 42.1 nm	
					T-Cel NPs: 439.0 ± 206.8 nm	
					P-Cel NPs: 874.9 ± 315.8 nm	
nanoparticles	self-assembly	erianin	celastrol: 96.45%	celastrol: 63.15%	CEN: 142.9 ± 2.75 nm (DLS)	[28]
			erianin: 82.61%	erianin: 37.85%		
nanoparticles	conjugation; self-assembly	galactose	NS	NS	CE-Gal-NPs: 130.15 ± 3.45 nm (DLS)	[29]
polymeric nanoparticles	precipitation; surface modification; sonication	chitosan	92.5%	82.4%	CNPs: 165.8 nm (DLS)	[35]
polymeric nanoparticles	chemical conjugation; dialysis	MAL, PEG, zein, AHP, TMC	75.26%	6.51%	Cel/AHP-NPs@TMC: 221.76 ± 6.73 nm (DLS)	[36]
polymeric nanoparticles	nano-precipitation; self-assembly	HA, zein	94.78%	2.63%	Cel/Zein@HA NPs: 156.04 ± 1.71 nm (DLS)	[37]
polymeric nanoparticles	self-assembly; nano-precipitation	PAPE, SA, *β*-CD	91.08%	6.75%	Cel/NPs: 50.04 ± 0.74 nm (DLS)	[38]
polymeric nanoparticles	saturated aqueous solution method	H4R6RGD, CM-*β*-CD, siPD-L1	NS	NS	H−C@C/siPD-L1 NPs: 200 nm	[39]
polymeric nanoparticles	chemical conjugation	Oxi-*β*-CD, FA, lecithin, PEG	NS	NS	NS	[53]
polymeric nanoparticles	chemical conjugation	TK, PEG, PLGA, FA	52.0%	10.8%	Cel-loaded PLGA-TK-PEG-FA NPs: 184 ± 1 nm	[54]
polymeric nanoparticles	polycondensation; emulsion solvent evaporation	PGA, DSPE-PEG	95.10%	4.53%	PGA@Cel NPs: 177.93 ± 4.72 nm (DLS)	[47]
polymeric nanoparticles	chemical conjugation; self-assembly	mPEG, BR	72.6%	6.6%	CLT/BRNP: 68.6 nm (DLS)	[48]
polymeric nanoparticles	ultrasonication-dialysis; injection-combined antisolvent precipitation	PEG	44.3%	77.8%	PEG/CSL: 125.4 ± 3.2 nm	[49]
		PEG-C18	83.7%	87.3%	PEG-C18/CSL: 121.7 ± 1.3 nm	
		TEG	26.9%	68.5%	TEG/CSL: 119.0 ± 0.9 nm	
polymeric nanoparticles	single emulsion; solvent evaporation	TPP, TPGS, PLGA, CS, FA	75.4%	36.1%	CS-FA/TT/PLGA@cela: 70–100 nm	[40]
polymeric nanoparticles	mechanical homogenization	PLGA	NS	NS	pCel: 7.2 µm	[41]
polymeric nanoparticles	emulsification; evaporation	PLGA, PEG, CS, MAN	57.7%	20.59%	Cel-NPs: 355.15 ± 15.25 nm (DLS)	[42]
polymeric nanoparticles	chemical conjugation; self-assembly	PLGA, *β*-CD, PEI, BM, Fc, LMWH	Fc: 98.56%	Fc: 3.08%	PP/H: 108.37 ± 1.02 nm	[43]
			Cela: 90.73%	Cela: 2.83%		
polymeric nanoparticles	emulsification; evaporation	PLGA, PEG, CS, MAN	67%	88%	Cel-NPs: 380 nm (DLS)	[44]
polymeric nanoparticles	flash nanoprecipitation	dextran, PLGA	88.1%	46.8%	FNP: 80–160 nm	[45]
polymeric nanoparticles	emulsion	PLGA, TG-18	NS	NS	TPL@NPs: 180 nm	[46]
					EL@NPs: 190 nm	
polymeric nanoparticles	dialysis	LMWH, PSN	28.25%	NS	PLC-NP: 115.83 ± 6.93 nm	[50]
polymeric nanoparticles	in situ chemical conjugation-induced self-assembly	mPEG-PLL	53.9%	9.95%	CEL-NPs: 65 ± 6.4 nm (DLS); 103.1 ± 10.7 nm (TEM)	[51]
polymeric nanoparticles	chemical conjugation; self-assembly	OPDMA, PHEMA	NS	42%	OPDMA-Cela: 100 nm	[55]
micelles	self-assembly	PEG, PCL	NS	7.36%	CNMs: 48 nm (DSL)	[112]
micelles	self-assembly	PEG, PPS	NS	4.71%	C-PEPS: 135 nm	[64]
micelles	chemical conjugation; self-assembly	PEG, PPS	56.24%	4.48%	Cel NMs: 56 nm (DLS)	[65]
micelles	chemical conjugation; thin film hydration	HA, FA, TPGS, PF127	89.52%	15.9%	CLS-HF/MLs: 120.6 ± 4.45 nm (DLS)	[71]
micelles	self-assembly	TPP, HA, Chol	97.21%	22.8%	Cst@HA/TPP-M: 131.4 ± 2.4 nm	[72]
micelles	self-assembly	TA, PEG, BA	NS	8.51%	C-PTEB: 70 nm	[67]
micelles	self-assembly	PEG, PCL, PEI	NS	6.58%	CPNM: 24.94 ± 5.42 nm (DLS)	[113]
micelles	self-assembly	PEG, PCL, PEI	NS	NS	NS	[114]
micelles	ultrasound emulsification	thermogelling dendronized chitosan	NS	2.1%	CMs: 13 nm (DLS)	[75]
micelles	self-assembly	Kolliphor HS 15, DC	NS	2.1%	DCCM: 15 nm (DLS)	[76]
micelles	ultrasound emulsification; thermogelling dendronized chitosan mix	chitosan	NS	NS	NS	[77]
micelles	chemical conjugation; dialysis	GA, CMCS, TK, Rh	87.91%	36.86%	Cela/GCTR PMs: 165.67 ± 2.27 nm (DLS)	[66]
micelles	thin-film hydration	OVA	99.89%	4.76%	OVA-NMs-celastrol: 50.72 ± 0.98 nm (DLS)	[74]
micelles	chemical conjugation; self-assembly	LU, CA, CS	86.9%	14.8%	Cel@LU-CA-CS: 95.0 ± 2.4 nm (DLS); 75.0 ± 5.0 nm (TEM)	[115]
micelles	chemical conjugation; emulsion solvent evaporation	GOC, BPOA	NS	NS	EDA-GLA/CE/2-PBA: 292.27 ± 13.52 nm (DLS)	[70]
micelles	chemical conjugation; self-assembly	EDA, GLA, 2-FPBA	90.10%	60.05%	Cel/GOC-2: 268.43 ± 0.93 nm	[68]
micelles	chemical conjugation; self-assembly	MET, CS, PBE	83.47%	14.60%	MET-CS-PBE@CLT: 200 nm (TEM)	[69]
micelles	self-assembly	mPEG, PCL, PEI, Nur77	NS	NS	PPP/Nur77/Cela: 100 nm (TEM)	[73]
dendrimers	chemical conjugation	EpCAM aptamer, PEG, G5-PAMAM dendrimer	55%	7.9% (Ce-G5-P-aEp)	Ce-G5-P-aEp: 265 ± 4.651 nm (DLS) Ce-G5-P-Ep: 231.4 ± 2.816 nm (DLS)	[79,80]
				16.3% (Ce-G5-P-Ep)		
dendrimers	chemical conjugation	glucose, PEG, IR808, TPP, G4-PAMAM, Azo	NS	NS	Glu-PEG-Azo/Mito-Cel808: 150~200 nm (DLS); 121.10 ± 11.08 nm (SEM)	[81]
lipid nanoparticles	solvent evaporation	VHPKQHRGGSKGC, EYL, soybean oil, DOTAP, DSPE, mPEG, MAL	0.87%	2.83%	PC-PLNs: 114.0 ± 1.85 nm (DLS)	[88]
lipid nanoparticles	ultrasonication	MO, P407	99.1%	NS	CS-LCNP: 194.1 ± 9.78 nm (DLS); 118–223 nm (TEM)	[89]
polymer-lipid nanoparticles	thin-film dispersion; emulsification	lecithin, cholesterol, stearylamine, CCm, HA	92.16%	3.7%	HMCLPs: 128.5 ± 5.84 nm (DLS)	[92]
polymer-lipid nanoparticles	double emulsion	ATRA, PLGA, PEG, DSPE, mPEG, lecithin	ATRA: 89.5%	ATRA: 9.5%	ATLP: 152.5 ± 1.26 nm (DLS)	[90]
			TRI: 82.1%	TRI: 8.4%		
polymer-lipid nanoparticles	solvent diffusion; in situ reduction	Se, SPC, PLGA	98.95%	17.07%	Se@Tri-PLNs: 123.1 nm (DLS)	[91]
protein-lipid nanoparticles	thin-layer evaporation	LEUKO	74.62% (C1)	3.73% (C1)	Cel@LEUKO (C1): 186.90 ± 2.16 nm	[93]
			73.98% (C2)	7.40% (C2)	Cel@LEUKO (C2): 208.10 ± 6.03 nm	
			49.06% (C3)	9.81% (C3)	Cel@LEUKO (C3): 227.07 ± 3.31 nm	
protein nanoparticles	high-pressure homogenization	BSA	75.0%	13.88%	Celastrol-BSA-NPs: 125.6 ± 2.2 nm (DLS)	[96]
protein nanoparticles	chemical conjugation; high-pressure homogenization	Lac-BSA	79.0%	13.62%	CEL-Lac-BSA: 158.6 ± 3.4 nm	[97]
protein nanoparticles	desolvation; ester condensation	RGD-BSA	75.8%	NS	CBR NPs: 144 ± 3.7 nm (DLS)	[98]
protein nanoparticles	high-pressure homogenization	LY, ^D^CDX-BSA	75%	NS	^D^CDX-BSA NPs: 126.8 nm (DLS)	[99]
protein nanoparticles	chemical conjugation; self-assembly	LF, DTX	NS	DTX: 6.94%	DTX-LF-CST: 157.8 nm (DLS); 115–132 nm (TEM)	[100]
				CST: 5.98%		
inorganic nanoparticles	metal coordination; self-assembly	Fe^3+^, TK-PEG, F127-PEI	NS	NS	CEL-Fe NPs: 86 nm	[104]
inorganic nanoparticles	metal coordination; self-assembly	Cu^2+^, DSPE-PEG	NS	NS	Cel-Cu NP: 110 nm (DLS)	[105]
inorganic nanoparticles	chemical conjugation	Au, PVP-co-2-dimethylaminoethyl methacrylate, FA	93.69%	49.97%	FCA: 22.00 ± 4.48 nm (TEM)	[116]
inorganic nanoparticles	double emulsion solvent evaporation	MIT, PEG-PLGA, CaCO_3_	NS	MIT: 21.6%	MCCaNPs: 98.0 ± 1.25 nm (DLS)	[111]
				CEL: 9.8%		
				CaCO_3_: 17.6%		
inorganic nanoparticles	gas diffusion; thin-film dispersion	CaCO_3_, GA, DSPE, PEG	NS	NS	GA-LCC-CEL: 206 ± 19 nm (DLS)	[117]
inorganic nanoparticles	co-precipitation	Ca_3_(PO_4_)_2_	75%	NS	CPCN: 140 ± 3 nm (DLS); 32 ± 5 nm (FESEM); 35 ± 5 nm (AFM)	[118]
inorganic nanoparticles	deprotonated coordination	MOF (ZIF-8): Zn^2+^, 2-MI, TEA, PVP	80%	NS	Cel-ZIF-8 NPs: 72 ± 1.7 nm (DLS)	[106]
inorganic nanoparticles	physical encapsulation	PEG-biotin, MOF (ZIF-8): Zn^2+^, 2-MI	60.52%	31.60%	CEL@ZIF-8@PEG-BIO: 234.5 nm (DLS)	[107]
inorganic nanoparticles	physical encapsulation	PEG-biotin, MOF (ZIF-8): Zn^2+^, 2-MI	60.74%	31.88%	CEL@ZIF-8@BIO: 262.6 nm (DLS)	[108]
inorganic nanoparticles	structural difference-based selective etching; simple diffusion	SiO_2_, CS, GPTMS	NS	24.3%	CSL@HMSNs-Cs: 290.17 nm (DLS); 260 nm (TEM)	[109]
inorganic nanoparticles	sol–gel	MSN, CA, N9, 8P	88.6%	8.14%	N9@M-CA~8P: 201.0 ± 1.8 nm (DLS); 60 nm (TEM); 60 nm (SEM)	[110]

NS: Not specified.

**Table 2:** Detailed description of examples of celastrol-based nanoparticle formulations in the treatment of diseases.

Indications	Models	Therapeutic Outcomes	Bioavailability	Pharmacokinetic Parameters	[Ref]
breast cancer	MCF-7 cells	reduced systemic toxicity; enhanced tumor accumulation; efficient tumor suppression	NS	NS	[27]
breast cancer	4T1 cells	effective killing of the entire tumor cell population; reduced toxic side effects; enhanced solubility; enhanced tumor-targeting capability	NS	NS	[28]
hepatocellular carcinoma	HepG2 cells	active targeting (Gal ligand) and passive targeting (EPR effect); improved drug targeting; superior in vivo tumor suppression; minimized systemic toxicity	NS	NS	[29]
	H22 cells				
breast cancer	MCF-7/ADR cells	elevated intracellular ROS levels for ERK/JNK signaling regulation; enhanced apoptosis induction of resistant cells; enhanced drug resistance reversal via HSF-1 activation and P-gp suppression	NS	NS	[35]
obesity	Caco-2 cells	enhanced cellular uptake; improved WAT vascular targeting; diminished systemic toxicity; active targeted delivery	NS	NS	[36]
hepatocellular carcinoma	HepG2 cells	promoted cell apoptosis via regulated Bax/Bcl-2 expression; suppressed tumor angiogenesis via down-regulated CD31 and VEGF; improved biosafety; enhanced tumor proliferation inhibition; diminished systemic toxicity	NS	t_1/2_ = 21.976 ± 0.923 h C_max_ = 32.780 ± 2.69 μg/mL AUC_0–t_ = 365.513 ± 28.07 μg/mL·h AUC_0–∞_ = 608.584 ± 41.92 μg/mL·h MRT_0–t_ = 9.226 ± 0.647 h CL = 0.004 ± 0.000 L/h/kg	[37]
ulcerative colitis	NCM460 cells	enhanced inhibition of M1 polarization and promotion of M2 polarization; suppressed inflammation via TLR4/NF-κB pathway inhibition; eliminated excessive ROS; preserved integrity of intestinal barrier; restored disordered gut microbiota	NS	NS	[38]
	RAW264.7 cells				
triple-negative breast cancer	MDA-MB-231 cells	enhanced cellular uptake and successful lysosomal escape for efficient intracellular siPD-L1 delivery; upregulated p53 to trigger Bax transcription; promoted endoplasmic reticulum CRT translocation; promoted tumor cell apoptosis	NS	NS	[39]
rheumatoid arthritis	RAW264.7 cells	enhanced cellular uptake by activated macrophages via FR-mediated endocytosis; induced macrophage apoptosis	NS	NS	[53]
ovarian cancer	SKOV3 cells	active tumor cell targeting; elevated intracellular ROS level to trigger tumor cell apoptosis	NS	NS	[54]
rheumatoid arthritis	MH7A cells	sustained drug release; enhanced cellular uptake; prolonged blood circulation; minimal side effects	NS	T_1/2_ = 10.05 ± 6.99 h AUC_0–t_ = 4839.09 ± 201.70 μg/mL·h MRT_0–∞_ = 13.54 ± 5.20 h	[47]
	RAW264.7 cells				
rheumatoid arthritis	RAW264.7 cells	ROS-triggered structural destruction via bilirubin oxidation; reduced pro-inflammatory cytokine secretion and suppressed of inflammation progression; reduced systemic toxicity	NS	NS	[48]
breast cancer	4T1 cells	altered carrier-drug interactions; varied drug release and antitumor efficacy	NS	NS	[49]
breast cancer	4T1 cells	IC_50_ (4T1) = 0.33 μg/mL enhanced cellular uptake; induced mitochondrial damage; upregulated apoptotic proteins; favorable biocompatibility; negligible systemic toxicity	NS	NS	[40]
neuropathic pain	NS	reduced ROS level, apoptosis, and inflammatory expression; significant suppressed glial activation; ROS scavenging and cytoprotective effects	NS	NS	[41]
acute lung injury; acute respiratory distress syndrome	RAW264.7 cells	suppressed NLRP3 inflammasome activation; upregulated LC3 autophagy protein; activated macrophages and neutrophils; absent hepatic toxicity	NS	NS	[42]
triple-negative breast cancer	4T1 cells	IC_50_ (4T1) = 20.37 ± 1.55 ng/mL elevated intracellular ROS; prominent tumor growth inhibition; improved in vivo biosafety	NS	NS	[43]
ulcerative colitis	RAW264.7 cells Caco-2 cells	mannose receptor-mediated macrophage uptake; inhibited NLRP3 inflammasome; suppressed pro-inflammatory cytokines; alleviated colonic mucosal injury; absent hepatic and renal toxicity	NS	NS	[44]
lung cancer	A549 cells	IC_50_ (A549) = 1.986 μg/mL selective inhibition; lowered hepatotoxicity; intracellular efficient delivery	NS	NS	[45]
rheumatoid arthritis	Jurkat cells RAW264.7 cells	IC_50_ (Jurkat) = 52.53 ng/mL; IC_50_ (RAW264.7) = 121 ng/mL synergistic anti-inflammatory effect; alleviated articular swelling and bone erosion; reduced systemic hepatotoxicity	NS	NS	[46]
primary/metastatic cancer	B16F10 cells	selectin-mediated tumor targeting; inhibited PI3K/Akt/mTOR pathway; reduced systemic hepatonephrotoxicity; enhanced in vivo anti-tumor and anti-metastasis efficacy	NS	NS	[50]
	4T1 cells				
	MCF-7 cells				
	MDA-MB-231 cells				
	A875 cells				
	A375 cells				
	SK-MEL-28 cells				
	SKBR-3 cells				
	A2058 cells				
melanoma	B16F10 cells	IC_50_ (B16F10) = 2.81 μM EPR-mediated tumor accumulation; enhanced anti-melanoma efficacy; alleviated hepatic and renal toxicity	NS	NS	[51]
breast cancer	4T1 cells	robust ICD induction; reduced PD-L1 expression; enhanced anticancer efficacy; reduced systemic toxicity	NS	NS	[55]
melanoma	B16F10 cells				
liver cancer	HepG2 cells				
cervix cancer	Hela cells				
lung cancer	A549 cells				
retinoblastoma	SO-Rb 50 cells	inhibited cell migration and invasion; suppressed overexpression of HIF-1α and VEGF-A; inhibited angiogenesis and vessel sprouting	NS	NS	[112]
rheumatoid arthritis	RAW264.7 cells	inhibited NF-κB and Notch1 pathways; suppressed M1 macrophage polarization and joint pathological injury	NS	NS	[64]
dry eye disease	HCE-2 cells	suppressed TLR4/MyD88/NF-κB pathway; inhibited corneal injury; protected goblet cells; promoted tear secretion; ROS-triggered drug release; low systemic toxicity	NS	NS	[65]
	CCL-20.2 cells				
breast cancer	MCF-7 cells	dual receptor-mediated tumor targeting; induced tumor apoptosis, cytoplasmic rupture, nuclear shrinkage, and tumor apoptosis	NS	t_1/2_ = 18.998 ± 0.766 h C_max_ = 35.800 ± 2.403 μg/mL AUC_0–∞_ = 469.370 ± 33.260 μg/mL·h MRT_0–∞_ = 22.898 ± 0.464 h Cl = 0.004 (mg/kg)/(μg/mL)/h Vss = 0.098 ± 0.005 (mg/kg)/(μg/mL)	[71]
	MDA-MB-231 cells				
triple-negative breast cancer	MDA-MB-231 cells	enhanced CD44-mediated cellular drug uptake; elevated intracellular ROS; accelerated cell apoptosis	NS	NS	[72]
retinoblastoma	Y79 cells	induced cell apoptosis; enhanced cellular uptake	NS	NS	[67]
corneal allograft rejection	corneal epithelial cells	downregulated TLR expression in M1 macrophages; inhibited TLR4/MyD88/NF-κB signaling pathway in macrophages; prolonged corneal allograft survival	NS	NS	[113]
corneal stromal fibrosis	corneal fibroblasts	suppressed TGF-β1/Smad2/3-YAP/TAZ signaling; downregulated expression of fibrotic proteins; alleviated postoperative corneal stromal fibrosis	NS	NS	[114]
posterior capsule opacification	lens epithelial cells	inhibited TGF-β1-induced lens epithelial cell proliferation; eliminated PCO; negligible ocular toxicity	NS	NS	[75]
corneal stromal fibrosis	primary corneal stromal cells	inhibited mTOR pathway via PI3K/AKT and Deptor signaling; inhibited TGF-β1-induced Smad2/3 phosphorylation; reduced extracellular matrix deposition and corneal scar formation	NS	NS	[76]
subconjunctival fibrosis	pterygium fibroblasts	inhibited TGF-β1-induced proliferation and migration of human pterygium fibroblasts; suppressed TGF-β1/Smad2/3-YAP/TAZ signaling cascade; reduced collagen deposition and subconjunctival fibrosis; Absence of obvious local toxicity	NS	NS	[77]
hepatocellular carcinoma	HepG2 cells	active hepatoma targeting; inhibited tumor cell proliferation with selectivity; low cytotoxicity against normal hepatocytes; inhibited in vivo tumor growth; low systemic organ toxicity	NS	t_1/2_ = 22.43 ± 1.33 h C_max_ = 1.31 ± 0.02 mg/L AUC_0–24h_ = 13.81 ± 0.08 mg/L·h AUC_0–∞_ = 21.43 ± 8.56 g/L·h MRT_0–24h_ = 10.37 ± 0.47 h MRT_0–∞_ = 26.41 ± 7.88 h V = 1.91 ± 0.63 L/kg CL = 0.10 ± 0.04 L/h/kg	[66]
	BEL-7402 cells				
allergic airway inflammation	NS	downregulated Th2 cytokines; elevation of antigen-specific IgG1/IgG2; alleviation of pulmonary inflammatory infiltration and allergic airway inflammation	NS	NS	[74]
acute kidney injury	HK-2 cells	inhibited NF-κB p65 and p38 MAPK inflammatory signaling pathway activation; ameliorated mitochondrial apoptosis pathway; upregulated anti-apoptotic Bcl-2; downregulated pro-apoptotic Cleaved Caspase-3; reduced inflammatory infiltration in renal tubules	NS	NS	[115]
psoriasis	HaCat cells	pH/GSH dual-responsive drug release; enhanced intracellular uptake by melanoma cells; inhibited proliferation with selectivity; negligible toxicity toward normal cells	NS	NS	[70]
melanoma	B16F10 cells	enhanced cellular and skin penetration; reduced cytotoxicity toward keratinocytes; alleviated psoriatic erythema and scaling	NS	NS	[68]
obesity	3T3-L1 cells	improved glucose and lipid metabolism; alleviated hepatic steatosis and adipose inflammation; minimal systemic organ toxicity	NS	NS	[69]
	RAW264.7 cells				
liver cancer	HepG2 cells	enhanced Nur77 mitochondrial translocation and Bcl-2 conformational conversion; elevated cytochrome c/caspase-9 expression; promoted tumor cell apoptosis and in vivo tumor suppression; negligible systemic organ toxicity	NS	NS	[73]
colorectal cancer	SW620 cells	EpCAM-targeted cellular uptake; enhanced cancer cell apoptosis; reduced hepatic and renal systemic toxicity; induced apoptosis via cleaved caspase-3 activation; reduced hepatotoxicity and nephrotoxicity	NS	NS	[79,80]
non-small cell lung cancer	A549 cells	enhanced cytotoxicity; induced apoptosis; inhibited metastasis; suppressed tumor proliferation and metastasis; low systemic toxicity	NS	NS	[81]
chronic kidney disease	glomerulus endothelial cells	effective inhibition of inflammation, reduction in endothelial damage, alleviation of CKD severity; increase in NO level via increasing eNOS while inhibiting VCAM-1 expression; improved glomerular pathology: reduced cellular proliferation, mesangial expansion, tubular necrosis, and IgA deposition	NS	NS	[88]
	podocyte cells				
asthma	BCi-NS1.1 cells	sustained drug release; anti-inflammatory activity via NF-κB pathway modulation	NS	NS	[89]
hepatocellular carcinoma	Hepa1-6 cells	elevated intracellular ROS and mitochondrial damage; promoted cell apoptosis; suppressed tumor proliferation; reduced systemic hepatonephric toxicity	NS	NS	[92]
inflammatory arthritis	RAW264.7 cells	inhibited pro-inflammatory cytokines (TNF-α, IL-6, IL-1β); suppressed NF-κB-regulated inflammatory responses; reduced histopathological features including cartilage destruction and bone erosion	NS	NS	[90]
enteritis (inflammatory bowel disease)	Caco-2 cells	higher cellular uptake; more potent in vivo anti-enteritis activity; resolved ulcerative colitis; modulated oxidative stress, inflammatory cytokines, and intestinal homeostasis	397%	C_max_ = 237.50 ± 26.82 ng/mL T_max_ = 7.33 ± 1.15 h AUC_0–∞_ = 8675.95 ± 322.14 ng/mL·h t_1/2_ = 13.77 ± 2.35 h	[91]
rheumatoid arthritis	RAW264.7 cells	inhibited inflammatory response via ROS-NF-κB pathway; effective targeting and transport of celastrol to inflammatory sites; inhibited ROS generation; reduced spleen index; alleviated liver injury	NS	NS	[93]
	MH7A cells				
inflammation; lipid accumulation	Caco-2 cells	better bioavailability and in vivo efficacy in obesity; reduced serum total cholesterol, AST, and ALT levels; endocytosis via clathrin-mediated pathway; anti-inflammatory M1→M2 skewing	NS	C_max_ = 24.20 ± 3.94 ng/mL AUC = 229.70 ± 32.33 ng/mL·h t_1/2_ = 7.83 ± 1.32 h MRT = 8.41 ± 0.16 h Clearance = 0.38 ± 0.07 L/h Vz = 4.24 ± 0.33 L	[96]
hepatic steatosis (non-alcoholic fatty liver disease)	HepG2 cells	reduced lipid deposition; ameliorated liver function; activated AMPK-SIRT1 pathway; downregulated FASN and SREBP1c; no major organ toxicity	NS	AUC = 37.44 ± 9.40 ng/mL·h	[97]
rheumatoid arthritis	neutrophils	induced INE apoptosis; inhibited NET release via NF-κB pathway and Cit-H3 reduction; reduced toxic effects; alleviated articular cartilage damage	NS	C_max_ = 163.274 ± 12.19 ng/mL AUC_0–t_ = 530.07 ± 41.47 μg/mL·h t_1/2_ = 25.29 ± 3.33 h	[98]
glioma	GL261 cells	BBB-penetrating and glioma-targeting via nAChRs and albumin-binding proteins; repolarized tumor-associated macrophages from M2 to M1 phenotype via STAT6 pathway suppression; blocked TGF-β/SMAD2 signaling pathway; enhanced apoptosis via caspase-3 upregulation	NS	NS	[99]
	C6 cells				
breast cancer	MCF-7 cells	inhibited in vivo tumor growth via downregulating NF-κB/TNF-α/COX-2 axis; reduced Ki-67 proliferation index in tumor tissues; enhanced tumor suppression	NS	NS	[100]
lung adenocarcinoma	A549 cells	IC_50_ (A549) = 6.96 μM IC_50_ (HepG2) > 16 μM IC_50_ (786-0) = 2.74 μM improved biosafety and bioavailability for cancer therapy; ROS-responsive drug release via thioketal group degradation; disrupted Hsp90-Cdc37 complex; downregulated Cdk4; induced apoptosis	NS	NS	[104]
hepatocellular carcinoma	HepG2 cells				
renal cell carcinoma	786-0 cells				
breast cancer	4T1 cells	induced self-amplified cuproptosis via coordination; scavenged GSH via NF-κB pathway; inhibited NF-κB and anti-tumor immunity	NS	NS	[105]
	MCF-7 cells				
	MDA-MB-231 cells				
	CAL-51 cells				
breast cancer	MCF-7 cells	high cellular uptake and significant tumor growth inhibition and apoptosis	NS	NS	[116]
colon cancer	CT26 cells	good blood biocompatibility; CRT translocation, HMGB1 and ATP release; significant tumor growth inhibition; increased intratumoral TNF-α, IFN-γ, and IL-12 secretion	NS	NS	[111]
breast cancer	4T1 cells	enhanced anti-proliferative and pro-apoptotic effects; modulated epithelial–mesenchymal transition protein expression; enhanced cellular uptake via endocytosis; upregulated Bax; downregulated Bcl-2	NS	NS	[117]
neuroblastoma	SH-SY5Y cells	protection against cell death; reduced intracellular ROS; reduced sub-G1 apoptotic cell population; protection against nuclear DNA damage	NS	NS	[118]
lung cancer	A549 cells	superior cytotoxicity; enhanced cell entry and protonation-triggered release; induced apoptosis via caspase-3/Bax/bcl-2 signaling pathway	NS	NS	[106]
ovarian cancer	SKOV3 cells	inhibited cell proliferation, MMP decline, and ROS generation; enhanced anti-tumor activity; up-regulated apoptosis-promoting biomarkers and P38/JNK MAPK signaling pathway; up-regulated Bax, cleaved caspase-7, and cleaved caspase-3; down-regulated Bcl-2; reduced hepatotoxicity and nephrotoxicity	NS	NS	[107]
	ES-2 cells				
colorectal cancer	HCT-116 cells	inhibited proliferation, G0/G1 cell cycle arrest; increased ROS production; reduced MMP; increased HO-1; decreased GPX4 and FTH1; inhibited tumor growth in vivo; reduced toxicity	NS	NS	[108]
	SW620 cells				
knee osteoarthritis	chondrocytes	improved articular surface erosion and joint effusion; inhibited upregulated expression of IL-1β, TNF-α, IL-6, MMP-3, MMP-13, and NF-κB signaling pathway; downregulated phosphorylated p65, IKKβ, and IκBα	NS	NS	[109]
cervical cancer	Hela cells	enhanced cytotoxicity; increased Cytochrome c release and PARP cleavage; no change in Bcl-2 expression level; suppressed in vivo tumor growth; good biosafety	NS	NS	[110]
colorectal cancer	LS174T cells				

NS: Not specified.

**Table 3:** Summary of recent celastrol-related studies on vesicle formulations.

Dosage Forms	Preparative Techniques	Composition	Encapsulation Efficiency	Loading Capacity	Particle Size	[Ref]
extracellular vesicles	sonication; encapsulation	BMSC-EVs; U-87 MG EVs; RAW264.6 EVs	72%	NS	BMSC-EVs-Cel: 150.6 ± 7.3 nm (DLS)	[143]
erythrocyte membrane vesicles	hypotonic & ultrasonic treatment; co-incubation	EMV	NS	NS	C-EMV: 245.61 ± 1.03 nm	[144]
liposomes	thin-film hydration	DSPC, Chol, DSPE, mPEG	74.45%	NS	TP lipo: 134.3 nm (DLS)	[150]
liposomes	lipid thin-layer-hydration-extrusion	Chol, SL, DSPE, PEG	78.77%	5.83%	Cel-LPs: 72.20 ± 27.99 nm (DLS)	[151]
liposomes	film dispersion; ultrasonication	PTX, soy lecithin, DSPE, PEG	CLT: 97.48%	7.79%	CP-Lip: 113.7 ± 6.13 nm (DLS); 100 nm (TEM)	[152]
			PTX: 86.29%			
liposomes	thin-film hydration	CREKA, DSPE, PEG	94.45%	4.72%	CREKA-Lip: 111.6 ± 1.5 nm (DLS)	[153]
liposomes	thin-film dispersion	MAN, PEG, DSPE, lecithin, Tween 80	90.8%	1.78%	CEL-MAN-LPs: 85.5 ± 0.7 nm (DLS)	[154]
liposomes	thin-film hydration	Chol, egg phosphatidylcholine, DSPE, PEG, MAN	92.19%	2.97%	MAN-CSL-lips: 95.37 ± 0.16 nm	[155]
liposomes	film dispersion	gala-PEG-DSPE, SPC, cholesterol	90.5%	NS	C-GPL: 139.4 ± 2.7 nm (DLS)	[156]
liposomes	dissolution; hydration; ultrasonication; filtration	*N*-octyl-*β*-D-glucopyranoside, cholesterol, soy bean phospholipid, DSPE, mPEG	NS	NS	AGCL: 83.41 ± 0.39 nm	[157]
liposomes	film dispersion	HA, SPC, cholesterol, TPP	98.37%	NS	C-TL/HA: 88.97 ± 1.27 nm (DLS)	[158]
liposomes	thin-film hydration; electrostatic layer-by-layer alternate deposition	pectin, TMC, EYL	94.45%	1.26%	Cel/PT-LbL Lipo: 179.95 ± 4.31 nm	[159]
liposomes	film dispersion	TET, coix seed oil	celastrol: 71.86%	celastrol: 37.921%	TET-CTM/L: 120 nm (DLS)	[160]
			tetrandrine: 98.45%	tetrandrine: 37.425%		
liposomes	modified ethanol injection	CuET, phospholipid	NS	NS	CuET-Lipid@Cela: 138.9 ± 1.3 nm (DLS)	[161]
exosomes	incubation; sonication	RSV, PEG, FA	NS	RSV: 19.61%	FA-Exo/R+C: 210.27 ± 7.27 nm (DLS)	[176]
				Cel: 0.37%		
exosomes	chemical conjugation	DSPE, PEG, KDEL, ME	CEL: 86.8%	CEL: 4.2%	KME: 155.7 ± 3.5 nm (DLS)	[177]
			siRNA: 79.5%	siRNA: 3.8%		
phytosomes	melting-hydration; in situ reduction	Se, SPC	98.85%	NS	Se@Tri-PTs: 126.7 nm (DLS)	[180]
phytosomes	melting-hydration; in situ reduction	Se, soybean lecithin	NS	NS	Se@Tri-PTs: 125 nm	[181]
phytosomes	NS	Se	NS	NS	NS	[182]
niosomes	thin-film hydration; sonication	Span 20, Span 60, cholesterol, carbopol	83.2%	3.33%	Cel Nio: 133 ± 2.1 nm	[186]

NS: Not specified.

**Table 4:** Detailed description of examples of celastrol-based vesicle formulations in the treatment of diseases.

Indications	Models	Therapeutic Outcomes	Bioavailability	Pharmacokinetic Parameters	[Ref]
glioblastoma	U-87 MG cells	inhibited tumor growth and neovascularization; modulated Akt and Caspase-3 expression; facilitated apoptosis; suppressed cell proliferation; reduced hepatotoxicity; alleviated renal toxicity	NS	NS	[143]
liver injury	bone marrow-derived macrophages	reduced serum ALT, AST, and BUN levels; inhibited pro-inflammatory M1 macrophage polarization; downregulated TNF-α, IL-1β, and iNOS expression; promoted anti-inflammatory M2 macrophage polarization; upregulated Arg-1, IL-10, and Ym-1 expression; reduced systemic hepatotoxicity	NS	NS	[144]
	RAW264.7 cells				
SARS (COVID-19)	Vero E6 cells	suppressed multiple pro-inflammatory cytokine release; downregulated NF-κB, p38 MAPK, and IL6/JAK/STAT3 inflammatory signaling pathways; upregulated anti-apoptotic p-STAT3 protein expression; improved pulmonary epithelial repair capacity; enhanced AT2-AM cell communication	NS	NS	[150]
rheumatoid arthritis	MH7A cells	dose- and time-dependent suppression of MH7A cell viability; sustained in vitro drug release especially under acidic pH environment; improvement in aqueous solubility and bioavailability; inhibition of inflammatory progression via NF-κB and Notch1 signaling pathways	5.78%	AUC_0–t_ = 711.27 ± 84.89 μg/L·h AUC_0–∞_ = 751.16 ± 76.94 μg/L·h MRT_0–t_ = 6.13 ± 1.03 h MRT_0–∞_ = 7.98 ± 0.33 h t_1/2_ = 11.71 ± 3.05 h T_max_ = 0.12 ± 0.06 h V_z/F_ = 44.63 ± 12.88 L/kg C_Lz/F_ = 2.71 ± 0.25 L/h/kg C_max_ = 372.40 ± 53.31 μg/L	[151]
pancreatic cancer	Pan02 cells	CI_50_ (Pan02) = 0.562 enhanced in vitro cell apoptosis; improved in vivo pharmacokinetic behaviors; elevated tumor inhibition rate; reduced systemic and cardiac toxicity	NS	AUC_0–t_ (CLT) = 18.620 ± 7.970 mg/L·h AUC_0–t_ (PTX) = 149.100 ± 64.815 mg/L·h AUC_0–∞_ (CLT) = 19.150 ± 7.910 mg/L·h AUC_0–∞_ (PTX) = 154.900 ± 64.468 mg/L·h t_1/2α_ (CLT) = 0.421 ± 0.121 h t_1/2α_ (PTX) = 0.138 ± 0.059 h t_1/2β_ (CLT) = 3.282 ± 1.27 h t_1/2β_ (PTX) = 1.470 ± 0.607 h CL_z_ (CLT) = 0.155 ± 0.016 L/h/kg CL_z_ (PTX) = 0.152 ± 0.008 L/h/kg C_max_ (CLT) = 5.049 ± 0.289 mg/L C_max_ (PTX) = 29.180 ± 8.848 mg/L	[152]
chronic kidney disease	NRK-49F cells	inhibited TGF-β1-induced fibrosis-related gene expression in activated cells; suppressed α-SMA, Col1a1, and fibronectin protein expression in renal fibroblasts; inhibited myofibroblast proliferation; downregulated PCNA, Cyclin E1, Cyclin D1, and Cyclin B1; reduced systemic toxicities including cardiotoxicity, hepatotoxicity, neurotoxicity, and reproductive toxicity	NS	T_1/2_ = 10.94 ± 0.50 h MRT_0–t_ = 10.74 ± 0.45 h C_max_ = 3.85 ± 0.78 mg/L AUC_0–t_ = 33.41 ± 6.53 mg/L·h	[153]
psoriasis	JAWS II cells	inhibited dendritic cell maturation; downregulated CD80, CD86, and MHC-II expression; suppressed mRNA expression of inflammatory cytokines IL-1β, IL-6, IL-17A, IL-23, and TNF-α in psoriatic skin lesions; reduced Ki67 expression in the basal layer of psoriatic epidermis	NS	NS	[154]
rheumatoid arthritis	RAW264.7 cells	suppressed pro-inflammatory cytokine secretion (TNF-α, IL-6, and IL-1β); inhibited osteoclast differentiation and formation; reduced systemic toxicity in liver, kidney, and spleen; inhibited osteoclast-mediated bone erosion via TRAP staining	NS	NS	[155]
liver cancer	HepG2 cells	suppressed AKT activation; downregulated p-AKT308 and p-AKT473; decreased liver damage biomarkers AST and ALT in serum; induced apoptosis; upregulated cleaved Caspase-3 and Caspase-3; retarded cell proliferation; reduced PCNA-positive cells; low systemic toxicity without severe weight loss and no histological damage to heart, lung, spleen, and kidney	NS	C_max_ = 6.42 ± 0.60 μg/mL AUC = 28.9 ± 1.13 μg/mL·h t_1/2_ = 4.06 ± 0.41 h MRT = 5.98 ± 0.79 h	[156]
hepatocellular carcinoma	SK-Hep1 cells	IC_50_ (HepG2) = 1.60–1.78 μM IC_50_ (SK-Hep1) = 0.85–1.12 μM induced mitochondrial apoptosis; upregulated cleaved Caspase-3 and Bax/Bcl-2 ratio; aggravated ROS generation and apoptotic body formation via VDAC2 knockdown; upregulated Caspase-3; downregulated Ki-67; reduced systemic toxicity	NS	NS	[157]
	HepG2 cells				
	H22 cells				
liver cancer	HepG2 cells	mitochondrial targeting via delivery into mitochondria; induced mitochondrial apoptosis pathway; upregulated Bax and Bak; downregulated Bcl-2; activated Caspase-9; reduced systemic toxicity	NS	NS	[158]
ulcerative colitis	NCM460 cells	suppressed pro-inflammatory cytokines IL-6, IL-1β, and TNF-α; improved colonic tissue penetration; inhibited TLR4/MyD88/NF-κB signaling pathway; reduced systemic toxicity	NS	NS	[159]
liver cancer	HepG2 cells	induced massive apoptosis; superior tumor accumulation via EPR effect; reduced systemic toxicity; inhibited CCL2 and TNF-α expression in serum; reduced CAFs; decreased α-SMA expression	NS	NS	[160]
cholangiocarcinoma	HuCCT1 cells	inhibited TMX2-mediated UPR activation; downregulated GRP78/BiP expression; downregulated TMX2 and GRP78/BiP expression; good safety; no obvious histological injury to major organs	NS	NS	[161]
sepsis	RAW264.7 cells	suppressed M1 macrophage polarization; downregulated iNOS expression; reduced F4/80^+^CD86^+^ cells; inhibited NF-κB and ERK1/2 signaling pathways; restored cardiac function	NS	NS	[176]
colon cancer	CT26 cells	ER-targeted delivery of PD-L1 siRNA; downregulated intracellular and membrane PD-L1 expression; inhibited tumor growth; elevated serum cytokines TNF-α and IL-6	NS	NS	[177]
colorectal adenocarcinoma	Caco-2 cells				
arthritis	Caco-2 cells	downregulated serum pro-inflammatory cytokines TNF-α, IL-6, and NO	NS	NS	[180]
inflammation	J774A.1 cells	inhibited NLRP3 inflammasome activation; suppressed caspase-1p20 and mature IL-1β release; inhibited NLRP3 inflammasome activation	NS	NS	[181]
podocyte injury in diabetic nephropathy	MPC-5 podocytes	reduced podocyte injury; restored autophagy activity; upregulated Beclin-1 and LC3-II/LC3-I ratio; reduced podocyte apoptosis	NS	NS	[182]
psoriasis	HaCaT cells	suppressed serum inflammatory cytokines IL-6, IL-17A, TNF-α, IL-2, and IFN-γ; inhibited inflammatory cytokine mRNA expression (IL-6, TNF-α, IFN-β); topical and systemic anti-psoriatic effect without systemic drug exposure	NS	NS	[186]

NS: Not specified.

**Table 5:** Summary of recent celastrol-related studies on emulsion formulations.

Dosage Forms	Preparative Techniques	Composition	Encapsulation Efficiency	Loading Capacity	Particle Size	[Ref]
nanoemulsions	ultrasonic emulsification	sesame oil, soybean lecithin, Pluronic F68	90%	NS	CEL NE: 91 nm	[203]
nanoemulsions	high pressure homogenisation	EYL, SO, MCT, DS, FH	91.7%	NS	SR-CLTNEs: 255.16 nm (DLS)	[204]

NS: Not specified.

**Table 6:** Detailed description of examples of celastrol-based emulsion formulations in the treatment of diseases.

Indications	Models	Therapeutic Outcomes	Bioavailability	Pharmacokinetic Parameters	[Ref]
melanoma	M10 cells	IC_50_ (M10, 48 h) = 4.5 μM IC_50_ (A375, 48 h) = 2.8 μM IC_50_ (B16F10, 48 h) = 3.0 μM IC_50_ (BPD6, 48 h) = 4.1 μM induced ICD; downregulated PD-L1 via TNF-α; increased infiltration of CD8^+^ T cells and NK cells; long-lasting immune memory	NS	AUC_0–72h_ = 12.2 ± 1.2 μg·h/mL T_1/2_ = 33.7 ± 0.5 h	[203]
	A375 cells				
	B16F10 cells				
	BPD6 cells				
rheumatoid arthritis	RAW264.7 cells	enhanced uptake by activated macrophages via SR-A mediation; protection against cartilage degradation; decreased serum levels of TNF-α and IL-1β; no significant hepatotoxicity or nephrotoxicity	NS	NS	[204]

NS: Not specified.

**Table 7:** Summary of recent celastrol-related studies on other formulations.

Dosage Forms	Preparative Techniques	Composition	Encapsulation Efficiency	Loading Capacity	Particle Size	[Ref]
carbon dots	hydrothermal method	PDA, CD	NS	NS	PDA-Ce-CDs: 260 nm	[209]
inclusion complexes	physical encapsulation; freeze-drying technique	SBE-*β*-CD	NS	NS	NS	[211]
hydrogels	physical encapsulation	lithocholic acid, dipeptide, doxorubicin, A13 hydrogelator	NS	NS	NS	[226]
hydrogels	physical encapsulation	carbomer 974	NS	NS	NS	[227]
hydrogels	physical encapsulation	GA, pectin	NS	70%	DN hydrogel: 21 ± 7 μm (SEM)	[221]
alloys	physical encapsulation	Mg, Al, LDH	NS	NS	NS	[228]
conjugates	chemical conjugation	CMCS	NS	1.5%	NS	[222]
conjugates	chemical conjugation	CSO	NS	10.19%	NS	[223]
conjugates	chemical conjugation	CSO	NS	10%	NS	[224]
collagen films	cross-linking	collagen, chitosan, glycerol or EDC-NHS	NS	NS	NS	[229]
microspheres	microfluidic electrospray	alginate hydrogel, soybean oil	86%	NS	NS	[217]
nanographene oxides	ultrasonication; chemical conjugation; physical encapsulation	nrGO, PEG	NS	75.22–92.18%	NS	[214]
nanotubes	self-assembly	BFP	88%	38%	BFP-Cel: 465.9 nm	[230]
microcapsules	physical encapsulation	porous starch, chitosan	68.7%	NS	NS	[218]
microneedles	micromolding	HA, ^TK^PF, pPUMA, HSA	80.6%	7.33%	CH NCs: 135.4 ± 2.1 nm (DLS)	[219]
microneedles	cross-linking	m*β*-CD, GelMA	NS	21.9%	NS	[220]
nanoporous membranes	electrospinning-coating	PLLA, HA	49.4%	26.79%	ONFM-CSR: 500–1200 nm (SEM)	[231]
magnetic microbubbles	emulsion solvent volatilization	C_3_F_8_, Fe_3_O_4_, CA, PEG, PLGA	83.1%	41.5%	Fe_3_O_4_-PEG2K-CST @PLGA: 298.1 nm	[212]
heterogeneous nanorods	rapid solution combustion	Fe_3_O_4_, α-Fe_2_O_3_, CA, PEG	96%	NS	Fe_3_O_4_/α-Fe_2_O_3_/CA-PEG-celastrol: 250–500 nm	[213]
nanosuspensions	antisolvent precipitation	P-188	98.18%	86.83%	CSL-NSps: 147.9 ± 2.16 nm (DLS); <100 nm (TEM)	[232]
nanosuspensions	precipitation; oxidative polymerization	PDA	NS	60.33%	PDA@CEL-NS: 189.67 ± 2.08 nm (DLS); ~100 nm (SEM)	[233]
microparticles	double emulsion solvent evaporation	PLGA, NaCl	72.8%	NS	Cela MPs: 2076 ± 390 nm	[216]
nano-patroller	one-pot annealing	ssDNA	NS	NS	tFNA: 60 nm	[225]

NS: Not specified.

**Table 8:** Detailed description of examples of other celastrol-based formulations in the treatment of diseases.

Indications	Models	Therapeutic Outcomes	Bioavailability	Pharmacokinetic Parameters	[Ref]
breast cancer	4T1 cells	excellent photothermal conversion ability; low hemolysis and good blood biocompatibility; high apoptotic rate; superior in vivo antitumor efficacy; low systemic toxicity	NS	NS	[209]
non-small cell lung cancer	A549 cells	IC_50_ (A549, 72 h) = 0.5 μM IC_50_ (H460, 72 h) = 0.6 μM improved intestinal permeability; enhanced anti-cancer efficacy	NS	t_1/2_ (PBS, pH7.4) = 71.9 ± 4.4 h t_1/2_ (rat plasma) = 65.1 ± 3.6 h t_1/2_ (human plasma) = 55.0 ± 4.7 h	[211]
	H460 cells				
colon cancer	CT26 cells	increased apoptosis; decreased proliferation at tumor site; overexpressed ceramide synthases (Cers1, Cers4, and Cers6); good biocompatibility	NS	NS	[226]
	HCT-8 cells				
	DLD-1 cells				
	HCT-116 cells				
psoriasis	Langerhans cells	reduced inflammatory cell infiltration and stratum spinosum thinning; reduced γδT cells and IL-17+ γδT cells; downregulated inflammatory factors (Il17a, Il1b, Il23a, S100a8, and Cxcl1); increased LC migration to draining lymph nodes; reduced monocytes and neutrophils in skin lesions	NS	NS	[227]
	γδT cells				
colon cancer	CT-26 cells	good biocompatibility and hemocompatibility; self-healing property; enhanced in vivo antitumor efficacy; reduced systemic toxicity; inhibited tumor angiogenesis; prolonged drug retention at tumor site	NS	NS	[221]
osteosarcoma	143b cells	enhanced corrosion resistance; inhibited bone tumor cell proliferation; induced apoptosis; inhibited PI3K-Akt-mTOR signaling pathway; inhibited osteoclast differentiation; good biosafety	NS	NS	[228]
	HOS cells				
obesity	BV2 cells	lower in vivo toxicity; reduced dietary intake; improved blood lipid profile; ameliorated lipid accumulation and adipocyte hypertrophy	NS	NS	[222]
	RAW264.7 cells				
pancreatic cancer	BxPC-3 cells	reduced cytotoxicity in hepatocytes; improved glucose homeostasis and insulin sensitivity; regulated adipocyte differentiation and lipid metabolism-related genes; lower systemic toxicity	NS	t_1/2_ = 8.97 ± 6.81 h T_max_ = 4.00 ± 0.00 h C_max_ = 32.39 ± 4.45 μg/mL AUC_0–t_ = 477.57 ± 48.70 h·μg/mL	[223]
obesity	L02 cells	reduced cytotoxicity in hepatic cells; inhibited cell migration and invasion; reduced in vivo toxicity; induced apoptosis in tumor tissue	NS	NS	[224]
periodontal disease	periodontal ligament fibroblasts	reduced TRAP-positive multinucleated osteoclasts; sustained morphology on collagen films	NS	NS	[229]
	bone marrow macrophages				
inflammatory pain	Caco-2 cells	alleviated inflammatory pain; dose-dependent analgesic effect; inhibited pro-inflammatory cytokines at mRNA level; high safety and low hepatotoxicity; gut-targeted release; protection from stomach acid	NS	NS	[217]
breast cancer	4T1 cells	synergistic chemo-photothermal cytotoxicity; reduced systemic toxicity	NS	NS	[214]
intrahepatic cholangiocarcinoma	HuCCT1 cells	liver targeting ability; enhanced anti-tumor efficacy; macrophage polarization from M2 to M1	NS	NS	[230]
colorectal cancer	Caco-2 cells	enhanced cellular uptake; enhanced bioavailability	NS	C_max_ = 486.78 ± 81.50 μg/L AUC_0–t_ = 4668.71 ± 637.97 μg/L·h	[218]
rheumatoid arthritis	RAW264.7 cells	induced apoptosis; upregulated PUMA; M1-to-M2 macrophage polarization; protection against bone erosion and cartilage damage; reduced pro-inflammatory cytokines in serum	NS	NS	[219]
skin infection	*Staphylococcus aureus*	disrupted MRSA biofilm; reduced inflammatory cytokines and M2 macrophage polarization; good biocompatibility and low hemolysis; increased collagen deposition; reduced inflammation in wound tissue	NS	NS	[220]
subconjunctival fibrosis	pterygium fibroblasts	inhibited TGF-β1/Smad2/3 signaling pathway; promoted LC3B expression in subconjunctival tissue; good biocompatibility and safety	NS	NS	[231]
liver transplant tumor	VX2 cells	low hemolysis; good biocompatibility; effective cellular uptake and lysosomal colocalization; enhanced apoptosis; targeted drug delivery and Fe_3_O_4_ accumulation; reduced systemic toxicity	NS	t_1/2_ = 26.16 ± 5.23 s AUC = 47.793 ± 18.48 mg/L·min	[212]
liver cancer	SMMC-7721 cells	enhanced cellular uptake under magnetic field; low cytotoxicity; decreased SOD activity; increased MDA level; upregulated Bax, caspase-3, and p53; downregulated Bcl-2; upregulated HIF-1α expression	NS	NS	[213]
breast cancer	4T1 cells	enhanced cytotoxicity; low systemic toxicity	NS	NS	[232]
liver cancer	HepG2 cells	IC_50_ (HepG2) = 2.238 μg/mL induced nuclear rupture and deformation; inhibited cell migration	NS	NS	[233]
malignant pleural mesothelioma	MSTO-211H cells	IC_50_ (MSTO-211H, 48 h) = 2.7 ± 0.3 µM IC_50_ (H28, 48 h) = 3.8 ± 0.6 µM IC_50_ (H2452, 48 h) = 3.8 ± 0.1 µM improved cytotoxicity; induced autophagy; upregulated LC3B; reduced cell viability	NS	NS	[216]
	H28 cells				
	H2452 cells				
obesity	NS	reduced hepatic fat deposition; M2 polarization of macrophages; reduced inflammation; reduced ER stress; increased STAT3 phosphorylation; good biocompatibility; no organ toxicity	NS	NS	[225]

NS: Not specified.

**Table 9:** Critical and comparative analysis between different delivery platforms.

Platforms	Advantages	Limitations	Technological Challenges	Translational Potential	[Ref]
polymeric nanoparticles	biodegradable, tunable release, protect labile drugs, surface functionalizable	potential burst release, polymer toxicity, batch-to-batch variability	controlled polymerization, scalable manufacturing, removal of residual solvents and organics	many approved products; strong for cancer and vaccines	[30,31,32,33,34]
lipid nanoparticles	high nucleic acid encapsulation, biocompatible, established for mRNA vaccines	oxidative degradation, hepatic accumulation, requires cold chain	stable size distribution, reproducible microfluidic mixing, targeting extrahepatic tissues	revolutionized mRNA delivery; expanding to gene editing, rare diseases	[82,83,84,85,86,87]
protein nanoparticles	biocompatible, bioactive, receptor-mediated uptake	immunogenicity risk, denaturation, enzymatic degradation	maintaining secondary structure, crosslinking control, large-scale GMP purification	limited by stability	[94,95]
inorganic nanoparticles	high stability, tunable shape and size, multi-modal imaging, photothermal potential	poor biodegradability, long-term toxicity, non-specific uptake	surface coating for colloidal stability, renal clearance regulation, off-target metal ion release	iron oxide approved; gold and silica in clinical trials	[101,102,103]
liposomes	mature technology, low toxicity, hydrophilic or hydrophobic drug loading	low drug loading, fast clearance without PEGylation, leakage	sterilization without degradation, scale-up (batch stability), controlling lamellarity	numerous FDA approvals; strong for chemotherapy, antifungals, and vaccines	[145,146,147,148,149]
exosomes	endogenous, low immunogenicity, cross biological barriers, natural targeting	low yield, heterogeneous cargo, difficult purification	scalable isolation, loading efficiency, regulatory definition	clinical trials; huge promise but industrial hurdles	[162,163,164,165,166,167,168,169,170,171,172,173,174,175]
phytosomes	enhanced bioavailability of phytoconstituents, phospholipid-based	poor stability in gastrointestinal fluids, batch variation from herbal extracts	standardization of active compounds, preventing aggregation, industrial drying methods	marketed supplements; limited by regulatory pathway for drugs	[178,179]
niosomes	non-ionic surfactants, cheaper and more stable than liposomes, easy to modify	surfactant toxicity, potential hemolytic activity, less characterized than liposomes	optimizing hydrophile-lipophile balance, avoiding vesicle fusion, sterilization	mostly cosmetic and dermatology; few clinical candidates	[183,184,185]
emulsions	high lipophilic drug loading, easy manufacturing, oral, intravenous, and ocular routes	thermodynamic instability (creaming and coalescence), large droplet size	preventing Ostwald ripening, surfactant selection, long-term shelf-life	well-established for parenteral nutrition	[187,188,189,190,191,192,193,194,195,196,197,198,199,200,201,202]
carbon dots	photoluminescence, small size, low cytotoxicity, high surface area	fluorescence quantum yield variability, poor understanding of biodegradation, liver accumulation	size- or shape-control via precursor and reaction conditions, purification from carbonaceous byproducts	mainly diagnostic and sensing; in vivo drug delivery early stage	[205,206,207,208]
inclusion complexes	enhanced solubility, protection from light or oxidation, FDA-approved	low drug loading, rapid drug release in blood	reducing renal toxicity, cost of modified cyclodextrins	approved formulations; mature but limited to small molecules	[210]

## Data Availability

No new data were created or analyzed in this study. Data sharing is not applicable to this article.

## Abbreviations

The following abbreviations are used in this manuscript:

ABPP	activity-based protein profiling
AI	artificial intelligence
ANX	Annexin
ATP	adenosine triphosphate
BBB	blood–brain barrier
BET	Brunauer–Emmett–Teller
BSA	bovine serum albumin
CRT	calreticulin
DC	dendritic cell
DLAT	dihydrolipoamide *S*-acetyltransferase
ELISA	enzyme-linked immunosorbent assay
EMA	European Medicines Agency
EpCAM	epithelial cell adhesion molecule
EPR	enhanced permeability and retention
FDA	Food and Drug Administration
FLS	fibroblast-like synoviocyte
GLUT	glucose transporter
GMP	Good Manufacturing Practice
GSH	glutathione
HA	hyaluronic acid
HMGB	high mobility group box
HSA	human serum albumin
ICD	immunogenic cell death
INE	inflammatory neutrophil
i.t.	intratumorally
IVIVC	in vitro-in vivo correlation
LMWH	low molecular weight heparin
LPS	lipopolysaccharide
MOF	metal–organic framework
NET	neutrophil extracellular trap
NF-κB	nuclear factor kappa-B
PAGE	polyacrylamide gel electrophoresis
PAMAM	poly(amidoamine)
PAT	process analytical technology
PCL	poly(*ε*-caprolactone)
PD	pharmacodynamics
PDA	poly(dopamine)
PD-L1	programmed cell death ligand 1
PEG	poly(ethylene glycol)
PEI	poly(ethylene imine)
PK	pharmacokinetics
PLGA	poly(lactic-*co*-glycolic acid)
PPS	poly(propylene sulfide)
PSN	P-selectin targeting peptide
PUMA	p53 upregulated modulator of apoptosis
QbD	quality-by-design
QC	quality control
ROS	reactive oxygen species
SDS	sodium dodecyl sulfate
siPD-L1	siRNA targeting programmed death ligand 1
tFNA	tetrahedral framework nucleic acid
^TK^PF	thioketal-crosslinked fluorinated polyethyleneimine
ZIF	zeolitic imidazolate framework
